# Retromer deficiency in Tauopathy models enhances the truncation and toxicity of Tau

**DOI:** 10.1038/s41467-022-32683-5

**Published:** 2022-08-27

**Authors:** Jamshid Asadzadeh, Evelyne Ruchti, Wei Jiao, Greta Limoni, Catherine MacLachlan, Scott A. Small, Graham Knott, Ismael Santa-Maria, Brian D. McCabe

**Affiliations:** 1 Brain Mind Institute, EPFL – Swiss Federal Institute of Technology Lausanne, Lausanne, Switzerland; 2 BioEM Facility, EPFL – Swiss Federal Institute of Technology Lausanne, Lausanne, Switzerland; 3 Department of Neurology, Columbia University, New York, USA; 4 Taub Institute for Research on Alzheimer’s Disease and the Aging Brain, Columbia University, New York, USA; 5 Department of Pathology & Cell Biology, Columbia University, New York, USA; 6 Facultad Ciencias Experimentales, Universidad Francisco de Vitoria, Pozuelo de Alarcón, Madrid, Spain

**Keywords:** Neurodegeneration, Molecular neuroscience

## Abstract

Alteration of the levels, localization or post-translational processing of the microtubule associated protein Tau is associated with many neurodegenerative disorders. Here we develop adult-onset models for human Tau (hTau) toxicity in *Drosophila* that enable age-dependent quantitative measurement of central nervous system synapse loss and axonal degeneration, in addition to effects upon lifespan, to facilitate evaluation of factors that may contribute to Tau-dependent neurodegeneration. Using these models, we interrogate the interaction of hTau with the retromer complex, an evolutionarily conserved cargo-sorting protein assembly, whose reduced activity has been associated with both Parkinson’s and late onset Alzheimer’s disease. We reveal that reduction of retromer activity induces a potent enhancement of hTau toxicity upon synapse loss, axon retraction and lifespan through a specific increase in the production of a C-terminal truncated isoform of hTau. Our data establish a molecular and subcellular mechanism necessary and sufficient for the depletion of retromer activity to exacerbate Tau-dependent neurodegeneration.

## Abstract (web summary)

Tau and the Retromer complex are both linked to Parkinson’s and Alzheimer’s disease. Using *Drosophila* neurodegeneration models, this study finds that low retromer activity induces a specific increase of a highly toxic truncated form of human Tau.

## Introduction

Tau, a microtubule-associated protein, stabilizes microtubule structures^1^ and is abundant in neural tissues^2,3^, predominantly within the axonal tracts of neurons^4^. Misfolded Tau species can contribute to insoluble aggregates that deposit within neurons in a class of neuropathologies known as Tauopathies^5^. Post-translational modified forms of Tau including hyperphosphorylation, as well as N- and C-terminal truncation, have been established as constituents of these pathological Tau deposits^6^ and these post-translational alterations can enhance both Tau tangle formation and fibrilogenicity^7,8^. A multitude of intracellular trafficking pathways are involved in the resolution and degradation of Tau aggregates^9–11^ and dysfunction of these pathways can exacerbate Tau accumulation and toxicity^12^.

The retromer complex is an evolutionarily conserved protein assembly that plays an essential role in the retrograde transport of proteins and associated lipids from endosomes to the trans-Golgi network and in the recycling of this cargo from endosomes back to the cell surface. The complex consists of a heterotrimeric protein core comprised of VPS26, VPS29, and VPS35^13–16^. This core assembly can interact with a variety of other intracellular trafficking proteins to regulate the transport of a wide range of cargo that enter the endosomal network either from the plasma membrane, via the biosynthetic or autophagic pathways, or from other sources. Mutations in retromer components are associated with familial Parkinson’s disease (PD)^17–22^ and defects in retromer activity are also linked with Alzheimer’s disease (AD)^23,24^. Consistent with an association of retromer with Alzheimer’s disease, retromer activity is reduced in the entorhinal cortex of late onset AD patients^25^. Decreasing retromer activity can inhibit long-term potentiation at hippocampal synapses^26^ and exacerbates memory deficits in a rodent AD model^27^. The molecular mechanisms through which retromer depletion may contribute to AD are still not fully understood. Retromer deficiency can alter Amyloid Beta (Aβ) levels^25,28,29^ and rodents deficient in retromer activity have increased levels of some species of Tau in the cerebrospinal fluid (CSF)^24^, similar to observations of Tau in the CSF of AD patients^24,30,31^.

Here, we have investigated the molecular and cellular mechanisms of the interaction of retromer with human Tau (hTau), using both in vivo *Drosophila* models and cell models of Tau toxicity. We find that reduction of retromer activity induces a potent enhancement of hTau toxicity through a specific and singular increase in the production of a C-terminal truncated isoform of hTau. We further demonstrate that interaction of the retromer complex with the late endosome protein Rab7 is critical for the enhancement of Tau-dependent neurodegeneration. Our data establish a molecular and cellular rationale causally linking deficient retromer activity with enhanced Tau toxicity.

## Results

### Expression of human Tau can disrupt development, reduce adult lifespan and induce axon retraction

**Fig. 1:**
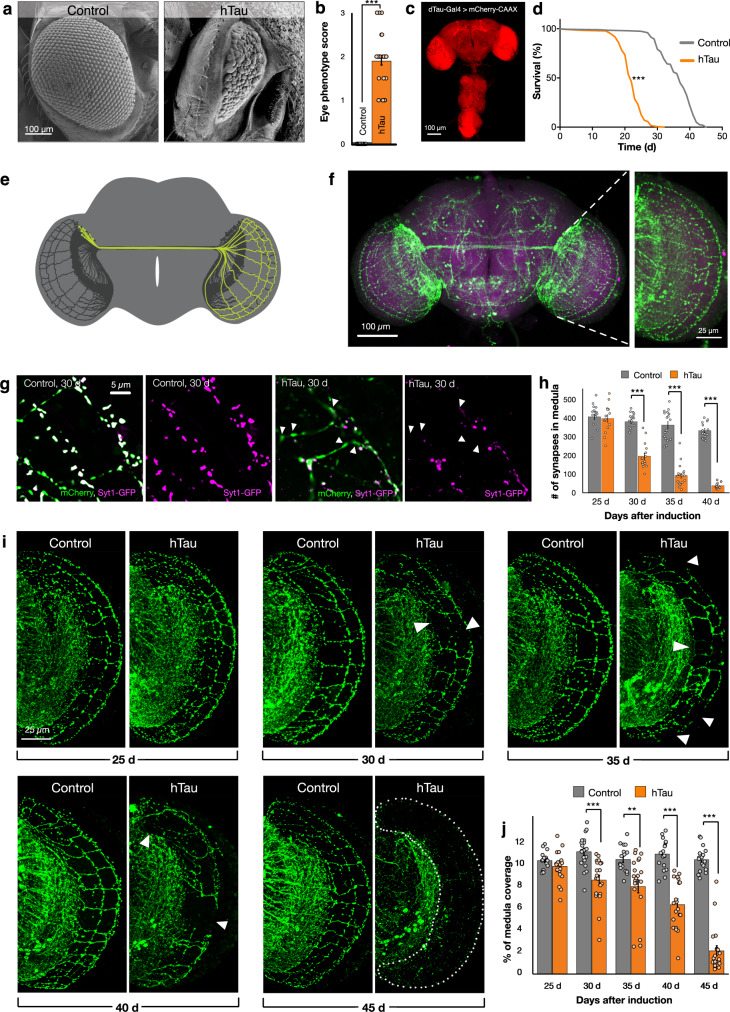
Expression of human Tau disrupts eye development, reduces adult lifespan, and induces synapse loss and axonal retraction. **a** Examples of eyes from control [GMR-Gal4, UAS-mCD8GFP] versus hTau expressing animals [GMR-Gal4, UAS-hTau, UAS-Control^Ri^]. **b** Eye disruption scores for indicated genotypes. Data are presented as mean values +/− SEM. **c** *Drosophila* adult brain expression of *dTau*-Gal4 labeled with UAS-mCherry-CAAX. **d** Survival analyses of control animals [*dTau*-Gal4, UAS-mCD8GFP, *tubulin*-Gal80^ts^] and hTau expressing adults [*dTau*-Gal4, UAS-hTau, *tubulin*-Gal80^ts^] **e** Schematic of the contralaterally projecting DC neurons subset of the *ato*-Gal4 line. **f** *ato*-Gal4 expressing neurons in adult *Drosophila* brain (left) with magnification of DC axonal subset in the medulla (right). **g** DC neuron medulla synapses labeled by Synaptotagmin-GFP [magenta] in control [*ato*-Gal4, UAS-Syt1-GFP, UAS-mCherry, UAS-mCD8GFP, *tubulin*-Gal80^ts^] and hTau expressing [*ato*-Gal4, UAS-hTau, UAS-Syt1-GFP, UAS-mCherry, *tubulin*-Gal80^ts^] animals, at 30 days after induction of transgene expression. In the control, axonal varicosities (mCherry) coincide with Syt1 labeling, in hTau expressing neurons many axonal varicosities lack Syt1 (arrowheads). **h** Quantification of the number of medulla synapses labeled by Synaptotagmin-GFP in control [*ato*-Gal4, UAS-Syt1-GFP, UAS-mCherry, UAS-mCD8GFP, *tubulin*-Gal80^ts^] and hTau [*ato*-Gal4, UAS-hTau, UAS-Syt1-GFP, UAS-mCherry, *tubulin*-Gal80^ts^] expressing animals in increments from 25 to 40 days after induction of transgene expression. Data are presented as mean values +/− SEM. **i** DC neuron axons in hTau [*ato*-Gal4, UAS-hTau, UAS-mCherry, *tubulin*-Gal80^ts^] and control animals [*ato*-Gal4, UAS-mCherry, UAS-mCD8GFP, *tubulin*-Gal80^ts^] from 25 to 45 days after induction of transgene expression. Gaps in axonal structure induced by hTau expression are indicated with arrowheads, when <4% axons are present, the medulla is outlined with dashed lines. **j** Quantification of medulla area occupied by DC neuron axons in hTau [*ato*-Gal4, UAS-hTau, UAS-mCherry, *tubulin*-Gal80^ts^] and control animals [*ato*-Gal4, UAS-mCD8GFP, UAS-mCherry, *tubulin*-Gal80^ts^]. Data are presented as mean values +/− SEM. Mann–Whitney test, ***p < 0.001 for eye disruption scores comparison between control (*n* = 32) and hTau (*n* = 34) (**b**). Mantel-Cox test, ***p < 0.001 for survival analysis of control (*n* = 202) vs. hTau (*n* = 192) (**d**), Mann–Whitney test, *p* = 0.701 for control (*n* = 18) vs. hTau (*n* = 15) (**h**, 25 days post induction), ***p < 0.001 for control (*n* = 18) vs. hTau (*n* = 16) (**h**, 30 days post induction), ***p < 0.001 for control (*n* = 17) vs. hTau (*n* = 19) (**h**, 35 days post induction), ***p < 0.001 for control (*n* = 16) vs. hTau (*n* = 15) (**h**, 40 days post induction). Mann–Whitney test, *p* = 0.1427 for control (*n* = 18) vs. hTau (*n* = 18) (**j**, 25 days post induction), ***p < 0.001 for control (*n* = 20) vs. hTau (*n* = 20) (**j**, 30 days post induction), **p = 0.0022 for control (*n* = 17) vs. hTau (*n* = 20) (**j**, 35 days post induction), ***p < 0.001 for control (*n* = 18) vs. hTau (*n* = 20) (**j**, 40 days post induction), ***p < 0.001 for control (*n* = 19) vs. hTau (*n* = 20) (**j**, 45 days post induction). *n* indicates independent biological replicates. Source data are provided as a Source Data file.

To begin to investigate the effects in *Drosophila* of expression of human Tau, we expressed a transgenic wildtype (WT) (2N4R) variant of hTau in the *Drosophila* eye^32^. As described previously^33^, expression of hTau during eye development induces aberrant morphology compared to controls which can be assigned a quantitative score (Fig. 1a, b and Supplementary Fig. 1a). Using these scoring criteria, eye morphology in hTau-expressing animals scored on average 1.89 AU (*p* < 0.001) compared to controls (0.04) (Fig. 1b), consistent with previous studies of hTau expression during development.

Having established expression of our hTau reagents could recapitulate prior observations during development, we next sought to determine the effects of hTau expression in adult animals in a physiologically appropriate pattern. To do this, we generated a Gal4 insertion in the endogenous *Drosophila* Tau (*dTau*) gene and coupled this with temporal control of expression only in adults^34^. We did note that *dTau* mRNA expression was reduced in this line relative to controls (Supplementary Fig. 1b), however even null mutants of *dTau* do not alter animal lifespan^35^. We confirmed using a reporter that *dTau*-Gal4 has broad neural expression in adult *Drosophila* similar to dTau protein expression^36^ (Fig. 1c). Using this line, we then expressed hTau only in adulthood using temperature-controlled induction beginning 3 days after adult emergence (eclosion). Compared to GFP expressing control animals, which had a median survival age of 37 days, we found animals expressing in hTau in adulthood had a 40.5% reduction (*p* < 0.001) in median survival to 22 days (Fig. 1d). Thus, expression of human Tau in adult *Drosophila* within the pattern of dTau expression dramatically reduces lifespan.

In numerous paradigms, increased levels of Tau result in the retraction and degeneration of axons^37^, but this is difficult to assay when expression patterns are broad, such as in *dTau*-Gal4. We therefore sought to develop an assay that would allow us to image with single axon resolution the effects of hTau expression on adult *Drosophila* neurons in vivo. To do this, we expressed hTau in a subset of adult neurons labeled by the *ato*-Gal4 line^38^ again using temperature-controlled induction to ensure expression only in adults. Neurons labeled by this line include a group of ~39 neurons^39^, designated Dorsal Cluster (DC) neurons, of which ~11 project contralateral axons to make stereotypical sparse synaptic connections forming a lattice in the optic medulla (Fig. 1e, f). This sparse and reproducible distribution allowed us to image individual axons and synapses in this brain region.

We first examined the synapses produced by DC neurons, labeled with Synaptotagmin-GFP (Fig. 1g), in control animals as they aged. We found that the number of DC neuron synapses was unchanged until 25 days after eclosion in control animals (Fig. 1h). After this age, there was a modest continual decrease in the number of DC neuron synapses such that 40-day old animals had on average 18% less (*p* < 0.001) synapses compared to 25-day old control animals (Fig. 1h). We then expressed hTau in these neurons beginning 3 days after adult eclosion. Similar to control animals, we observed no change in the number of DC neuron synapses during the first 25 days of hTau expression. However, after this age, we observed a rapid loss of DC neuron medulla synapses in hTau expressing animals (Fig. 1g, h) with a 49% (*p* < 0.001) decrease in synapse numbers at 30 days, a 75% (*p* < 0.001) decrease after 35 days and an 89% (*p* < 0.001) decrease after 40 days compared to control animals of the same age. Therefore, expression of hTau in adult DC neurons results in a progressive loss of synaptic connections.

We next examined if this loss of synapses was accompanied by changes in axons of DC neurons in the medulla during adult lifespan. Unlike DC neuron synapses, we found no change in the area occupied by DC neuron axons within the medulla through lifespan in control animals (Fig. 1i, j). When hTau was expressed in DC neurons, we also observed no change in the medulla area occupied by these axons for the first 25 days after hTau expression (Fig. 1i, j). However, beginning at 30 days, the axons in hTau expressing animals became obviously perturbed with noticeable axonal loss (Fig. 1i, j) evidenced by a 23% (*p* < 0.001) reduction in DC neuron medulla axonal area compared to controls. Axonal loss was coincident with the thinning and fragmentation of the remaining axons (Supplementary Fig. 1c). Progressively more DC neuron axons were lost in hTau expressing animals as they continued to age (Fig. 1i, j), until only 20% (*p* < 0.001) of DC medulla axons were present compared to controls at 45 days after the induction of hTau expression. The retraction and loss of DC neuron axons was not accompanied by cell death of DC neurons however, as the number of DC neurons in hTau expressing animals did not decline compared to controls at any age timepoint (Supplementary Fig. 1d–f). To determine if axons of DC neurons were also perturbed when hTau was broadly expressed, we labeled DC neurons with *ato*-lexA^39^ while simultaneously expressing hTau with *dTau*-Gal4. We also observed a loss of DC neuron axons in the context of broad hTau expression (Supplementary Fig. 1g), similar to that seen when hTau was expressed with *ato*-Gal4, consistent with axon loss as a recurrent outcome of hTau expression in neurons. In summary, expression of human Tau in *Drosophila* adults reduces lifespan and induces an age-dependent progressive loss of synapses and retraction of axons.

### Inhibition of retromer enhances the toxicity of human Tau

We next sought to examine the effects of inhibiting retromer components on hTau-mediated toxicity in each of the Tau models described above. We decided to employ transgenic RNAi (^Ri^) constructs to allow tissue-specific knockdown of VPS26, VPS29, and VPS35 proteins. We first validated the efficiency of these constructs to reduce retromer protein levels (Supplementary Fig. 2a, b). We observed an 86% reduction of VPS26 (*p* < 0.001), an apparently complete inhibition of VPS29 expression (*p* < 0.001), and a 41% reduction of VPS35 (*p* < 0.02) using these lines.

**Fig. 2:**
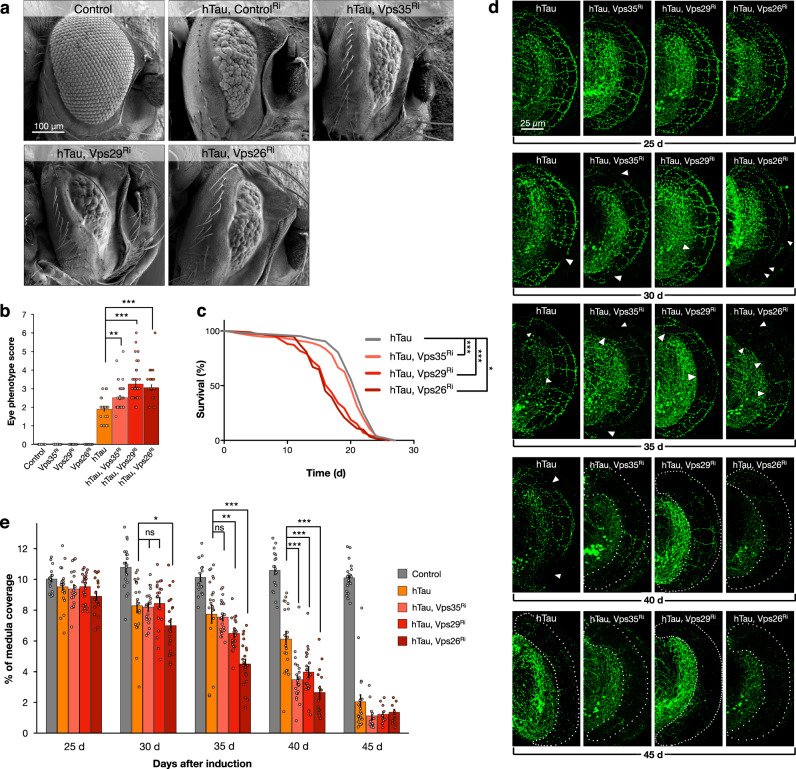
Retromer depletion exacerbates the toxicity of human Tau. **a** Eye morphology in control [GMR-Gal4, UAS-mCD8GFP], hTau expression together with control RNAi [GMR-Gal4, UAS-hTau, UAS-GFP^Ri^] and hTau expression [GMR-Gal4, UAS-hTau] together with RNAi inhibition of retromer components (plus UAS-Vps35^Ri^ or UAS-Vps29^Ri^ or UAS-Vps26^Ri^]. **b** Eye disruption scores for indicated genotypes. Data are presented as mean values +/− SEM. **c** Survival analyses of hTau [*dTau*-Gal4, UAS-hTau, UAS-Cherry^Ri^, *tubulin*-Gal80^ts^] and hTau expressing adults [*dTau*-Gal4, UAS-hTau, *tubulin*-Gal80^ts^] together with retromer inhibition [plus UAS-Vps35^Ri^ or UAS-Vps29^Ri^ or UAS-Vps26^Ri^]. **d** DC neuron medulla axons with hTau expression alone [*ato*-Gal4, UAS-hTau, UAS-mCherry, UAS-mCD8GFP, *tubulin*-Gal80^ts^] and hTau expression [*ato*-Gal4, UAS-hTau, UAS-mCherry, *tubulin*-Gal80^ts^] together with retromer inhibition [UAS-Vps35^Ri^ or UAS-Vps29^Ri^ or UAS-Vps26^Ri^] from 25 to 45 days after onset of transgene expression. Gaps in axonal structure induced by hTau expression are indicated with arrowheads, when <4% axons are present, the medulla is outlined with dashed lines. **e** Quantification of medulla area occupied by DC neuron axons with hTau expression alone [*ato*-Gal4, UAS-hTau, UAS-mCherry, UAS-mCD8GFP, *tubulin*-Gal80^ts^] and hTau expression [*ato*-Gal4, UAS-hTau, UAS-mCherry, *tubulin*-Gal80^ts^] together with retromer inhibition [UAS-Vps35^Ri^ or UAS-Vps29^Ri^ or UAS-Vps26^Ri^] from 25 to 45 days after onset of transgene expression. Data are presented as mean values +/− SEM. Kruskal–Wallis test, ***p < 0.001, for eye disruption scores across all genotypes with multiple comparisons between hTau (*n* = 34) and hTau plus Vps35^Ri^ (*n* = 38) (Dunnett’s test **p = 0.003), hTau and hTau plus Vps29^Ri^ (*n* = 38) (Dunnett’s test ***p < 0.001), and hTau and hTau plus Vps26^Ri^ (*n* = 29) (Dunnett’s test ***p < 0.001). *n* = 23 for control, *n* = 31 for Vps35^Ri^, *n* = 30 for Vps29^Ri^ and *n* = 31 for Vps26^Ri^ (**b**). Mantel-Cox test, ***p = 0.0005 for survival analysis of hTau (*n* = 148) vs. hTau plus Vps35^Ri^ (*n* = 153), ***p < 0.001 for hTau vs. hTau plus Vps29^Ri^ (*n* = 126), and ***p < 0.001 for hTau vs. hTau plus Vps26^Ri^ (*n* = 173) (**c**). Kruskal-Wallis test, *p* = 0.4212 (**e**, 25 days post induction) for hTau (*n* = 21) vs. hTau plus Vps35^Ri^ (*n* = 20) (Dunnett’s test *p* = 0.9855), hTau vs. hTau plus Vps29^Ri^ (*n* = 24) (Dunnett’s test *p* = 0.9999), hTau vs. hTau plus Vps26^Ri^ (*n* = 18) (Dunnett’s test *p* = 0.2582), *n* = 18 for control. Kruskal-Wallis test, *p = 0.0351 (**e**, 30 days post induction) for hTau (*n* = 20) vs. hTau plus Vps35^Ri^ (*n* = 19) (Dunnett’s test *p* = 0.9974), hTau vs. hTau plus Vps29^Ri^ (*n* = 20) (Dunnett’s test *p* = 0.988), hTau vs. hTau plus Vps26^Ri^ (*n* = 20) (Dunnett’s test *p = 0.0476), *n* = 20 for control. Kruskal–Wallis test, ***p < 0.001 (**e**, 35 days post induction) for hTau (*n* = 20) vs. hTau plus Vps35^Ri^ (*n* = 20) (Dunnett’s test *p* = 0.6797), hTau vs. hTau plus Vps29^Ri^ (*n* = 20) (Dunnett’s test **p = 0.0091), and hTau vs. hTau plus Vps26^Ri^ (*n* = 22) (Dunnett’s test ***p < 0.001), *n* = 17 for control. Kruskal-Wallis test, ***p < 0.001 (**e**, 40 days post induction) for hTau (*n* = 20) vs. hTau plus Vps35^Ri^ (*n* = 22) (Dunnett’s test ***p < 0.001), hTau vs. hTau plus Vps29^Ri^ (*n* = 20) (Dunnett’s test ***p = 0.0005), and hTau vs. hTau plus Vps26^Ri^ (*n* = 15) (Dunnett’s test ***p < 0.001), *n* = 18 for control. Kruskal–Wallis test, *p* = 0.2815 (**e**, 45 days post induction) for hTau (*n* = 20) vs. hTau plus Vps35^Ri^ (*n* = 11) (Dunnett’s test *p* = 0.1407), hTau vs. hTau plus Vps29^Ri^ (*n* = 19) (Dunnett’s test *p* < 0.1209), and hTau vs. hTau plus Vps26^Ri^ (*n* = 11) (Dunnett’s test *p* = 0.1728), *n* = 19 for control. n indicates independent biological replicates. Source data are provided as a Source Data file.

We next assessed the effects of knock-down using these lines upon eye morphology. We found that consistent with previous reports^40,41^, inhibition of retromer components did not induce aberrant eye development (Fig. 2b and Supplementary Fig. 2c). We then combined inhibition of retromer with these constructs together with simultaneous expression of human Tau. When hTau was expressed while VPS26, VPS29, or VPS35 were inhibited, we found a significant increase of the disruption of eye morphology compared to hTau expression alone (Fig. 2a, b) (+37% [*p* < 0.001] hTau & Vps26^Ri^ vs hTau, +41% [*p* < 0.001] hTau & Vps29^Ri^ vs hTau, and +24% [*p* < 0.003] hTau & Vps35^Ri^ vs hTau). The magnitude of the enhancement we observed was consistent with the relative efficiency of RNAi inhibition (Supplementary Fig. 2a, b). These results suggested that reduction of retromer activity could enhance the toxicity of Tau at least during eye development.

To determine if this interaction also occurred in adult neurons, we next inhibited retromer components while co-expressing hTau. We first examined the effects of retromer inhibition alone upon lifespan when expressed using *dTau*-Gal4. We observed that inhibition of VPS29 did not significantly alter median lifespan (36 days Vps29^Ri^ vs 37 days control^Ri^, [*p* = 0.8]), however, inhibition of VPS26 did significantly reduce median lifespan by 5.4% (*p* < 0.001, 35 days Vps26^Ri^ vs 37 days control^Ri^), while VPS35 inhibition reduced median lifespan by 19% (*p* < 0.001, 30 days Vps35^Ri^ vs 37 days control^Ri^) (Supplementary Fig. 2d). We then co-expressed these constructs together with hTau in adults. We found that inhibition of retromer components further reduced median survival compared to hTau expression alone (Fig. 2c), with VPS26 inhibition reducing lifespan by an additional 27% (*p* < 0.001, 16 days hTau & Vps26^Ri^ vs hTau 22 days), VPS29 inhibition reducing lifespan by an additional 23% (*p* < 0.001, 17 days hTau & Vps29^Ri^ vs hTau 22 days), and VPS35 inhibition reducing median lifespan by a further 9% (*p* < 0.001, 20 days hTau & Vps35^Ri^ vs hTau 22 days). Since VPS35 inhibition alone can significantly alter median lifespan, it is difficult to conclude if the combination with hTau is additionally more detrimental in this assay, however, the pronounced effect of VPS29 inhibition (which does not alter lifespan in controls), and VPS26 inhibition (which reduces lifespan fivefold more in the presence of hTau than in controls) was consistent with an enhancement of hTau toxicity when retromer activity is inhibited.

Building upon this finding, we then examined the effects of retromer depletion upon hTau-dependent axonal retraction in adult neurons. We found no alteration of the area occupied by DC neuron axons within the medulla when VPS26, VPS29, or VPS35 were inhibited alone in these neurons throughout lifespan (Supplementary Fig. 2e). We then coupled retromer inhibition with expression of hTau in DC neurons. While axonal retraction did not initiate at an earlier timepoint, we did observe a significant enhancement of axon loss (Fig. 2d, e) in these animals beginning 35 days of age. Inhibition of VPS26 increased axon loss by +15% (*p* < 0.047, hTau & Vps26^Ri^ vs hTau) at 30 days, though inhibition of VPS35 and VPS29 did not significantly increase axonal loss at this age. Inhibition of VPS26 increased axon loss by 41% (*p* < 0.001, hTau & Vps26^Ri^ vs hTau) and inhibition of VPS29 by +16% (*p* < 0.01, hTau & Vps29^Ri^ vs hTau) at 35 days though inhibition of VPS35 did not significantly increase axonal loss at this age. However, by 40 days, inhibition of VPS35 did significantly increase axonal loss by 43% (*p* < 0.001, hTau & Vps35^Ri^ vs hTau) as continually did inhibition of VPS26 (+56.7%, *p* < 0.001) and VPS29 (+35%, *p* < 0.001). This axonal loss progressed to 45 days such that only 12.1% (*p* < 0.001) of axons were retained on average when any component of the retromer complex was inhibited compared to controls (Fig. 2d, e). Therefore, as with eye morphology and longevity, retromer inhibition also enhanced the retraction of axons in response to the expression of human Tau. We also questioned if oppositely increasing retromer expression could modulate Tau-dependent axonal degeneration. However, we found that increasing the expression of Vps26, Vps29 or Vps35 alone in DC neurons using transgenes induced considerable axonal loss (Supplementary Fig. 2f) precluding additional interaction experiments. It is noteworthy that increased expression of retromer components also induces neurodegeneration in mice^42^. In summary, depletion of retromer activity, which does not compromise axonal integrity alone, does exacerbate the Tau-dependent neurotoxicity.

### Retromer inhibition increases Tau truncation

**Fig. 3:**
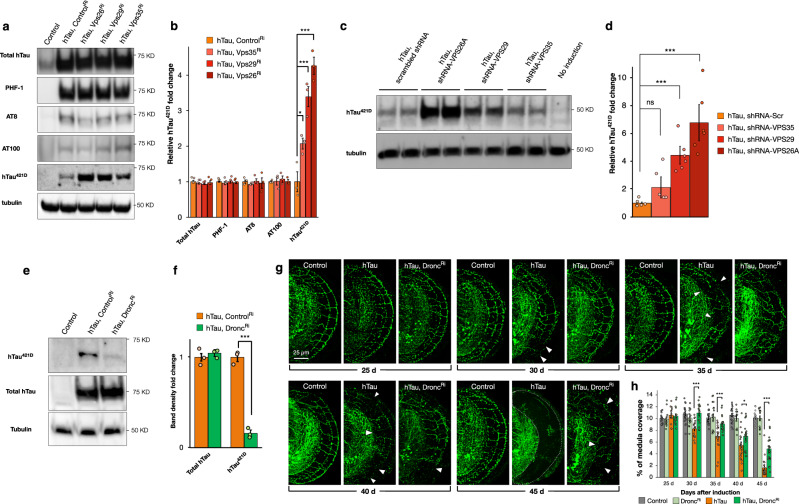
Human Tau truncation requires the caspase Dronc. **a** hTau protein analyses from head lysates of controls [GMR-Gal4, UAS-mCD8GFP], hTau expressing animals [GMR-Gal4, UAS-hTau, UAS-GFP^Ri^], and hTau [GMR-Gal4, UAS-hTau] expression together with retromer inhibition [UAS-Vps35^Ri^ or UAS-Vps29^Ri^ or UAS-Vps26^Ri^]. **b** Quantification of total Tau protein levels, Tau phosphorylation (PHF-1, AT8, and AT100) or truncation (Tau^421D^). Data are presented as mean values +/− SEM. **c** Protein analyses of truncated Tau levels in CN1.4 mouse cortical neurons expressing inducible full-length human Tau (0N4R) and scrambled shRNA or shRNAs against murine VPS26A, or VPS29, or VPS35. **d** Quantification of truncated Tau levels in CN1.4 neurons expressing human Tau and scrambled shRNA or shRNAs against murine VPS26A, or VPS29, or VPS35. Data are presented as mean values +/− SEM. **e** Protein analyses of head lysates from control [GMR-Gal4, UAS-mCD8GFP], hTau expressing [GMR-Gal4, UAS-hTau, UAS-GFP^Ri^] animals, and hTau expression together with Dronc inhibition [GMR-Gal4, UAS-hTau, UAS-Dronc^Ri^], probed with anti-hTau^421D^ (top) or anti-total Tau [TauC] (middle). **f** Quantification of total and truncated Tau levels from RNAi control [GMR-Gal4, UAS-hTau, UAS-GFP^Ri^] animals and hTau expression together with Dronc inhibition [GMR-Gal4, UAS-hTau, UAS-Dronc^Ri^]. Data are presented as mean values +/− SEM. **g** DC neuron medulla axons in control [*ato*-Gal4, UAS-mCherry, UAS-mCD8GFP, *tubulin*-Gal80^ts^], hTau expressing [*ato*-Gal4, UAS-hTau, UAS-mCherry, UAS-mCD8GFP, *tubulin*-Gal80^ts^] animals, and hTau expression together with Dronc inhibition [*ato*-Gal4, UAS-hTau, UAS-Dronc^Ri^, UAS-mCherry, *tubulin*-Gal80^ts^] from 25 to 45 days after onset of transgene expression. **h** Quantification of medulla area occupied by DC neuron control [*ato*-Gal4, UAS-mCherry, UAS-mCD8GFP, *tubulin*-Gal80^ts^], hTau expressing [*ato*-Gal4, UAS-hTau, UAS-mCherry, UAS-mCD8GFP, *tubulin*-Gal80^ts^] animals, and hTau expression together with Dronc inhibition [*ato*-Gal4, UAS-hTau, UAS-Dronc^Ri^, UAS-mCherry, *tubulin*-Gal80^ts^] from 25 to 45 days after onset of transgene expression. Data are presented as mean values +/− SEM. One-Way ANOVA, *p* = 0.5466, *n* = 3 for all genotypes (**b**, total hTau), One-way ANOVA, *p* = 0.8216, *n* = 3 for all genotypes (**b**, PHF-1), One-way ANOVA, *p* = 0.8216 *n* = 3 for all genotypes, (**b**, PHF-1), One-way ANOVA, *p* = 0.8791, *n* = 3 for all genotypes (**b**, AT8), One-Way ANOVA, *p* = 0.8982, *n* = 3 for all genotypes (**b**, AT100), One-way ANOVA, ***p < 0.001, *n* = 3 for all genotypes (**b**, hTau^421D^) for hTau (*n* = 3) vs. hTau plus Vps35^Ri^ (*n* = 3) (Dunnett’s test *p = 0.0361), hTau vs. hTau plus Vps29^Ri^ (*n* = 3) (Dunnett’s test ***p = 0.001), and hTau plus Vps26^Ri^ (*n* = 3) (Dunnett’s test ***p = 0.001). One-way ANOVA, *p* < 0.001 (**d**) for hTau plus scrambled (*n* = 3) vs. hTau plus shRNA-VPS35 (*n* = 3) (Dunnett’s test *p* = 0.4188), hTau plus scrambled vs. hTau plus shRNA-VPS29 (*n* = 3) (Dunnett’s test **p = 0.32), and hTau plus scrambled vs. hTau plus shRNA-VPS26 (*n* = 3) (Dunnett’s test ***p < 0.001). Unpaired two-tailed *t*-test, *p* = 0.5215, (**f**, total hTau) for hTau (*n* = 3) vs. hTau plus Dronc^Ri^ (*n* = 3), unpaired two-tailed *t*-test, *p* < 0.001, (**f**, hTau^421D^) for hTau (*n* = 3) vs. hTau plus Dronc^Ri^ (*n* = 3). Mann–Whitney test, *p* = 0.4071 for hTau (*n* = 16) vs. hTau plus Dronc^Ri^ (*n* = 16) (**h**, 25 days post induction, *n* = 18 for control, *n* = 16 for Dronc^Ri^), Mann–Whitney test, ***p < 0.001 for hTau (*n* = 20) vs. hTau plus Dronc^Ri^ (*n* = 18) (**h**, 30 days post induction, *n* = 20 for control, *n* = 15 for Dronc^Ri^), Mann–Whitney test, ***p < 0.001 for hTau (*n* = 20) vs. hTau plus Dronc^Ri^ (*n* = 20) (**h**, 35 days post induction, *n* = 17 for control, *n* = 18 for Dronc^Ri^), *p = 0.0396 for hTau (*n* = 20) vs. hTau plus Dronc^Ri^ (*n* = 20) (**h**, 40 days post induction, *n* = 18 for control, *n* = 13 for Dronc^Ri^), ***p < 0.001 for hTau (*n* = 20) vs. hTau plus Dronc^Ri^ (*n* = 20) (**h**, 45 days post induction, *n* = 19 for control, *n* = 15 for Dronc^Ri^). *n* indicates independent biological replicates. Source data are provided as a Source Data file.

We next sought to interrogate potential mechanisms through which retromer depletion could increase the toxicity of Tau. Alteration of the phosphorylation status of Tau has been linked to increased toxicity^43,44^ so we first compared the phosphorylation status of hTau in vivo using the phosphospecific antibodies PHF-1, AT8, and AT100^45^ when hTau was expressed alone or when retromer activity was simultaneously reduced. However, we found no change in the phosphorylation state of Tau when retromer activity was depleted as compared to hTau expression alone with these reagents (Fig. 3a, b). Truncated isoforms of Tau have become increasingly appreciated as important pathogenic contributors to Tau toxicity^7,8,46–49^. We therefore probed for truncated Tau using an antibody specific to hTau truncated at Aspartate 421 [hTau^421D^]^46^. In stark contrast to measurements of Tau phosphorylation, we observed a dramatic 4.2-fold increase (*p* < 0.001) in the levels of hTau^421D^ when VPS26 was inhibited together with hTau expression (Fig. 3a, b). This increase was also observed when VSP29 (3.4-fold [*p* < 0.001], hTau & Vps29^Ri^ vs hTau) or VPS35 (2.0-fold [*p* < 0.05], hTau & Vps35^Ri^ vs hTau) were inhibited when compared to hTau expression alone (Fig. 3a, b).

To validate the specificity of the effect of retromer depletion upon Tau truncation, we sought to rescue inhibition of *Drosophila* retromer activity with human transgenes for VPS26A, VPS29, and VPS35. We generated *Drosophila* transgenes for human retromer components, which due to nucleotide sequence differences, are resistant to inhibition by *Drosophila* RNAi constructs. We then simultaneously co-expressed these human transgenes together with hTau and the RNAi construct targeting the orthologous *Drosophila* retromer gene. We found that co-expression of hVPS26A, hVPS29 or hVPS35 significantly reduced the production of truncated hTau^421D^ when *Drosophila* retromer components were inhibited (Supplementary Fig. 3a, b). These results confirmed the specificity of retromer inhibition in *Drosophila* to enhance the levels of truncated human Tau.

We next sought to determine if the effect of retromer depletion on the levels of truncated human Tau was reproducible in an independent human Tau model system. To do this, we utilized a mouse cortical neuron cell line that enables the inducible expression of human Tau^50^ (Supplementary Fig. 3c). Induction of hTau expression in these cells, either alone or together with lentivirus expression of scrambled shRNA constructs results in low levels of hTau^421D^ production (Fig. 3c and Supplementary Fig. 3c). However, when murine retromer components were additionally inhibited by lentivirus shRNA expression (Supplementary Fig. 3d), we observed either a ~7-fold or a 4.5-fold increase in hTau^421D^ production for mVPS26A or mVPS29, respectively. Levels of hTau^421D^ were also increased when mVPS35 was inhibited, however, this did not reach statistical significance due to the higher variability of this shRNA construct. In sum, depletion of retromer activity in either *Drosophila* or murine neuronal cells results in a large increase in the levels of truncated human Tau.

### Inhibition of the endosomal caspase Dronc reduces Tau truncation and toxicity

In mammalian neurons, Tau can be truncated at Aspartate 421 by the activity of Caspases 3, 7, or 8^46^. To determine the *Drosophila* Caspase that can cleave human Tau, we employed transgenic RNAi to inhibit the *Drosophila* caspases Drice, Dredd, and Dronc in animals expressing hTau and measured the levels of hTau^421D^ produced. We found that inhibition of the caspases Drice and Dredd did not reduce the levels of hTau^421D^ (Supplementary Fig. 3e, f). In contrast, inhibition of the caspase Dronc^51^ strongly reduced (~6 fold, *p* < 0.001) the levels of hTau^421D^ while the total amount of hTau protein was unaffected (Fig. 3e, f). Dronc, which is present in endosomes^52^, in addition to roles in apoptosis^53,54^ has also recently been shown to have non-apoptotic functions^55–58^. Therefore, we tested if inhibition of Dronc could also ameliorate the elevated levels of hTau^421D^ produced when retromer activity is reduced. Inhibition of Dronc when VPS26 was depleted in the presence of hTau eliminated the increased levels of hTau^421D^ (Supplementary Fig. 3g, h) compared to expression of hTau with Vps26 inhibition alone. To further confirm that Dronc could cleave hTau, we also overexpressed a GFP-tagged Dronc construct together with hTau and measured hTau^421D^ levels. Dronc overexpression induced a significant increase in hTau^421D^ levels (>2-fold, *p* < 0.001, Supplementary Fig. 3i, j) confirming the ability of Dronc to truncate hTau. Therefore, hTau cleavage at Aspartate 421, with or without retromer inhibition, requires the activity of the caspase Dronc.

Expanding upon the finding that Dronc is necessary for hTau cleavage at 421D in *Drosophila*, we next asked if inhibiting this cleavage could alter the toxicity of hTau. When broadly expressed in the adult brain with *dTau*-Gal4, Dronc RNAi expression alone greatly reduced lifespan (−18.9%, *p* < 0.001, −7 days survival median Dronc^Ri^ vs control^Ri^) compared to control animals (Supplementary Fig. 3k). Nonetheless, Dronc inhibition in hTau-expressing adults was modestly beneficial resulting in a small, but significant, elongation of lifespan by 4.3% ([*p* < 0.001], +1 day median survival hTau & Dronc^Ri^ vs hTau & control^Ri^) (Supplementary Fig. 3l).

We then tested the effects of inhibiting Dronc in DC neurons expressing hTau. Axons of DC neurons expressing Dronc^Ri^ alone were unperturbed similar to controls (Fig. 3h). When Dronc was inhibited together with hTau expression however, this strongly reduced the toxicity of hTau in these neurons, delaying the onset of axonal retraction at Day 30 (32% more axons than hTau alone) such that at this age there was no significant difference to controls (Fig. 3g, h). The relative preservation of axons persisted throughout lifespan so that at 45 days, three fold more (*p* < 0.001) axons were preserved in the medulla when Dronc was simultaneously inhibited compared to hTau expression alone (Fig. 3g, h). These results demonstrate that the caspase cleavage of Tau is an important contributor to toxicity in these models, suggesting that the accumulation of truncated Tau when retromer is reduced may be essential for exacerbation of Tau toxicity.

### Increased production of truncated Tau by retromer inhibition is essential for enhanced toxicity

**Fig. 4:**
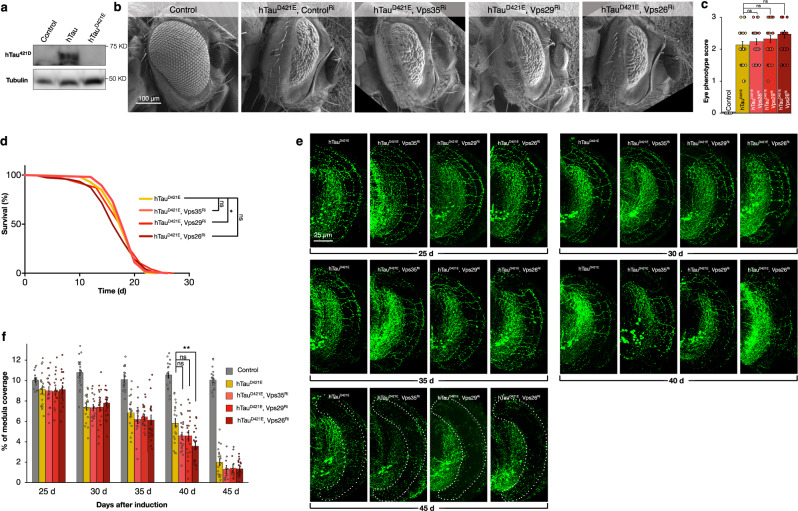
Abolishing Tau truncation abrogates enhancement of toxicity by retromer inhibition. **a** Protein analyses of Tau^421D^ truncated isoform levels in control [GMR-Gal4, UAS-mCD8GFP], hTau expressing [GMR-Gal4, UAS-hTau] and hTau^D421E^ mutant expressing [GMR-Gal4, UAS-hTau^D421E^] animals. **b** Eye morphology of control [GMR-Gal4, UAS-mCD8GFP], hTau^D421E^ expressing [GMR-Gal4, UAS-hTau^D421E^, UAS-GFP^Ri^], and hTau^D421E^ [GMR-Gal4, UAS- hTau^D421E^] expression plus inhibition of retromer components [UAS-Vps35^Ri^ or UAS-Vps29^Ri^ or UAS-Vps26^Ri^] animals. **c** Eye disruption scores for control [GMR-Gal4, UAS-mCD8GFP], hTau^D421E^ expressing [GMR-Gal4, UAS-hTau^D421E^, UAS-GFP^Ri^] and hTau^D421E^ [GMR-Gal4, UAS- hTau^D421E^] expression plus inhibition of retromer components [UAS-Vps35^Ri^ or UAS-Vps29^Ri^ or UAS-Vps26^Ri^] animals. Data are presented as mean values +/− SEM. **d** Survival analyses of hTau^D421E^ expressing [dTau-Gal4, UAS-hTau^D421E^, UAS-Cherry^Ri^, *tubulin*-Gal80^ts^] and hTau^D421E^ [*dTau*-Gal4, UAS-hTau^D421E^, *tubulin*-Gal80^ts^] expression together with retromer inhibition [UAS-Vps35^Ri^ or UAS-Vps29^Ri^ or UAS-Vps26^Ri^] adults. **e** Example DC neuron medulla axons in hTau^D421E^ expressing alone [*ato*-Gal4, UAS-hTau^D421E^, UAS-mCherry, UAS-mCD8GFP, *tubulin*-Gal80^ts^], and hTau^D421E^ expressing [*ato*-Gal4, UAS-hTau^D421E^, UAS-mCherry, *tubulin*-Gal80^ts^] together with retromer inhibition [UAS-Vps35^Ri^ or UAS-Vps29^Ri^ or UAS-Vps26^Ri^], from 25 to 45 days after onset of transgene expression. Gaps in the axonal structures are indicated with arrowheads. In brains with <4% axons, the medulla is outlined with broken lines. **f** Quantification of medulla area occupied by DC neuron axons in control [*ato*-Gal4, UAS-mCherry, UAS-mCD8GFP, *tubulin*-Gal80^ts^], hTau^D421E^ expressing alone [*ato*-Gal4, UAS-hTau^D421E^, UAS-mCherry, UAS-mCD8GFP, *tubulin*-Gal80^ts^], and hTau^D421E^ expressing [*ato*-Gal4, UAS-hTau^D421E^, UAS-mCherry, *tubulin*-Gal80^ts^] together with retromer inhibition [UAS-Vps35^Ri^ or UAS-Vps29^Ri^ or UAS-Vps26^Ri^]. Data are presented as mean values +/− SEM. Kruskal–Wallis test, *p* = 0.3272 for eye disruption scores across all genotypes with multiple comparisons between hTau^D421E^ (*n* = 33) and hTau^D421E^ plus Vps35^Ri^ (*n* = 30) (Dunnett’s test *p* = 0.1716), hTau^D421E^ and hTau^D421E^ plus Vps29^Ri^ (*n* = 31) (Dunnett’s test *p* = 0.5443), and hTau^D421E^ and hTau^D421E^ plus Vps26^Ri^ (*n* = 29) (Dunnett’s test *p* = 0.9899), *n* = 32 for control (**c**). Mantel-Cox test, *p* = 0.2508 for survival analysis of hTau^D421E^ (*n* = 248) vs. hTau^D421E^ plus Vps35^Ri^ (*n* = 218), *p = 0.0105 for hTau^D421E^ vs. hTau^D421E^ plus Vps29^Ri^ (*n* = 233), and *p* = 0.0988 for hTau^D421E^ and hTau^D421E^ plus Vps26^Ri^ (*n* = 260) (**d**). Kruskal–Wallis test, *p* = 0.9847 (**f**, 25 days post induction) with multiple comparisons between hTau^D421E^ (*n* = 20) and hTau^D421E^ plus Vps35^Ri^ (*n* = 19) (Dunnett’s test *p* = 0.9873), hTau^D421E^ and hTau^D421E^ plus Vps29^Ri^ (*n* = 20) (Dunnett’s test *p* = 0.9557), and hTau^D421E^ and hTau^D421E^ plus Vps26^Ri^ (*n* = 18) (Dunnett’s test *p* = 0.9999), *n* = 18 for control. Kruskal–Wallis test, *p* = 0.9552 (**f**, 30 days post induction), with multiple comparisons between hTau^D421E^ (*n* = 19) and hTau^D421E^ plus Vps35^Ri^ (*n* = 16) (Dunnett’s test *p* = 0.9991), hTau^D421E^ and hTau^D421E^ plus Vps29^Ri^ (*n* = 20) (Dunnett’s test *p* = 0.9999), and hTau^D421E^ plus Vps26^Ri^ (*n* = 18) (Dunnett’s test *p* = 0.9714), *n* = 20 for control. Kruskal-Wallis test, *p* = 0.4127 (**f**, 35 days post induction), with multiple comparisons between hTau^D421E^ (*n* = 18) and hTau^D421E^ plus Vps35^Ri^ (*n* = 16) (Dunnett’s test *p* = 0.5464), hTau^D421E^ and hTau^D421E^ plus Vps29^Ri^ (*n* = 20) (Dunnett’s test *p* = 0.8007), and hTau^D421E^ plus Vps26^Ri^ (*n* = 19) (Dunnett’s test *p* = 0.4417), *n* = 17 for control. Kruskal–Wallis test, *p = 0.0127 (**f**, 40 days post induction), with multiple comparisons between hTau^D421E^ (*n* = 19) and hTau^D421E^ plus Vps35^Ri^ (*n* = 20) (Dunnett’s test *p* = 0.108), hTau^D421E^ and hTau^D421E^ plus Vps29^Ri^ (*n* = 19) (Dunnett’s test *p* = 0.111), and hTau^D421E^ plus Vps26^Ri^ (*n* = 19) (Dunnett’s test **p = 0.0013), *n* = 18 for control. Kruskal–Wallis test, *p* = 0.4553 (**f**, 45 days post induction), with multiple comparisons between hTau^D421E^ (*n* = 20) and hTau^D421E^ plus Vps35^Ri^ (*n* = 11) (Dunnett’s test *p* = 0.2311), hTau^D421E^ and hTau^D421E^ plus Vps29^Ri^ (*n* = 13) (Dunnett’s test *p* = 0.3127), and hTau^D421E^ plus Vps26^Ri^ (*n* = 20) (Dunnett’s test **p = 0.1683), *n* = 19 for control. n indicates independent biological replicates. Source data are provided as a Source Data file.

To further interrogate if the truncation of Tau is pivotal for the enhanced toxicity when retromer activity is reduced, we generated a transgenic mutant form of hTau where we changed the codon for Aspartate 421 to Glutamate (hTau^D421E^), in an approach that has been previously demonstrated to abolish caspase cleavage of hTau at this amino acid^46^. As expected, when we probed for truncated hTau in animals expressing this modified Tau construct, no hTau^421D^ was detectable (Fig. 4a). We then used this hTau^D421E^ transgene to test interactions with retromer. When expressed in the eye, similar to wild-type hTau (hTau^WT^) hTau^D421E^ also disrupted morphology (Fig. 4b, c). However, unlike hTau^WT^ expressing animals, when retromer components were simultaneously depleted in animals expressing hTau^D421E^, we observed no enhancement of toxicity when either VPS35 or VPS29 were inhibited (Fig. 4b, c). We next evaluated the interaction of retromer depletion with hTau^D421E^ on adult lifespan using dTau-Gal4 expression. Similar to hTau^WT^, hTau^D421E^ expressing animals have 51.3% (*p* < 0.001) shorter median lifespan compared to controls. However, unlike hTau^WT^, concomitant knock-down of VPS26, VPS29 or VPS35 together with hTau^D421E^ expression did not additionally shorten lifespan (Fig. 4d).

We next expressed hTau^D421E^ in adult DC neurons. Again, similar to expression of hTau^WT^, expression of hTau^D421E^ in adult DC neurons induced progressive loss of axons over time (Fig. 4e, f). However, simultaneous inhibition of VPS26 and VPS29 did not accelerate or increase this loss at any time point. This was also the case for all timepoints when VPS26 was inhibited together with hTau^D421E^ expression, apart from measurements at 40 days after induction, where we did observe a small but significant additional loss of axons when VPS26 was inhibited (Fig. 4e, f), though the magnitude of this loss was less (18% less, *p* < 0.01) than that observed with hTau^WT^ expression (Fig. 2d, e). Therefore, not only does depletion of retromer activity induce a large accumulation of truncated human Tau, but inhibition of the production of this truncated isoform, either by mutating the Tau cleavage site or by inhibiting the essential caspase, abrogates the increased toxicity of Tau when retromer is reduced.

**Fig. 5:**
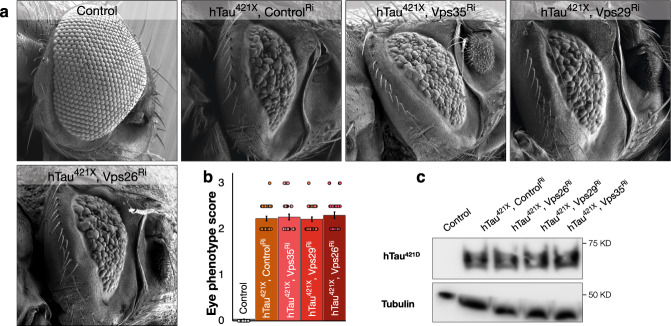
The toxicity of extant truncated Tau is not enhanced by retromer depletion. **a** Eyes from control [GMR-Gal4, UAS-mCD8GFP], hTau^421X^ expressing [GMR-Gal4, UAS-hTau^421X^, UAS-GFP^Ri^] animals, and hTau^421X^ [GMR-Gal4, UAS-hTau^421X^] expressing together with retromer inhibition [UAS-Vps35^Ri^ or UAS-Vps29^Ri^ or UAS-Vps26^Ri^] animals. **b** Eye disruption scores for control [GMR-Gal4, UAS-mCD8GFP], hTau^421X^ expressing [GMR-Gal4, UAS-hTau^421X^, UAS-GFP^Ri^] animals, and hTau^421X^ [GMR-Gal4, UAS-hTau^421X^] expressing together with retromer inhibition [UAS-Vps35^Ri^ or UAS-Vps29^Ri^ or UAS-Vps26^Ri^] animals. Data are presented as mean values +/− SEM. **c** Truncated Tau (hTau^421D^) levels from head lysates of control [GMR-Gal4, UAS-mCD8GFP], hTau^421X^ expressing [GMR-Gal4, UAS-hTau^421X^, UAS-GFP^Ri^], and hTau^421X^ expressing [GMR-Gal4, UAS-hTau^421X^] together with retromer inhibition [UAS-Vps35^Ri^ or UAS-Vps29^Ri^ or UAS-Vps26^Ri^] animals. Kruskal–Wallis test, *p* = 0.8297 for eye disruption scores across all genotypes with multiple comparisons between hTau^421X^ (*n* = 30) and hTau^421X^ plus Vps35^Ri^ (*n* = 30) (Dunnett’s test *p* = 0.8779), hTau^421X^ and hTau^421X^ plus Vps29^Ri^ (*n* = 32) (Dunnett’s test *p* = 0.9999), and hTau^421X^ and hTau^421X^ plus Vps26^Ri^ (*n* = 31) (Dunnett’s test *p* = 0.564). *n* indicates independent biological replicates. Source data are provided as a Source Data file.

Conceivably, the increased accumulation of hTau^421D^ that we observe when retromer function is inhibited could be the result of an increase in the production of truncated Tau molecules or from a failure to appropriately process and degrade existing truncated Tau species. To differentiate between these models, we next generated *Drosophila* lines that express hTau with a stop codon after Aspartate 421 [hTau^421X^] so the entirety of hTau in these animals was truncated. Consistent with prior observations, we found this truncated isoform of hTau was highly toxic^8,47^, so for interaction studies we generated transgenic lines that produce lower levels of hTau^421X^ than the equivalent full length hTau lines. Nonetheless, this lower amount of transgenic hTau^421X^ expression resulted in a strong disruption of eye morphology equivalent to expression of higher levels of full length hTau (Fig. 5a). In these animals, we then inhibited retromer activity. We found that in contrast to animals expressing full length hTau, we observed no increased disruption of eye morphology when we inhibited VPS35, VPS29 or VPS35 in the context of hTau^421X^ expression (Fig. 5a, b). Furthermore, when we examined the levels of truncated Tau protein present in these animals, we found no increase when retromer was inhibited compared to transgenic hTau^421X^ expression alone (Fig. 5c). These data are consistent with the reduction of retromer activity increasing the production of truncated Tau from full length Tau rather than a reduced ability to remove this truncated species.

### Diminished interaction of Retromer with Rab7 leads to an accumulation of truncated Tau in late endosomes

**Fig. 6:**
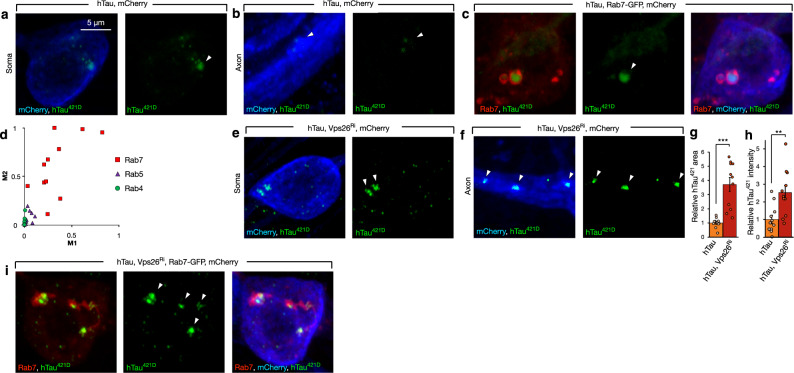
Reduction of Retromer leads to accumulation of truncated Tau in late endosomes. **a**, **b** Left panel: Soma and axons of dHB9 neurons expressing hTau and mCherry (blue) [*dHB9*-Gal4, UAS-hTau, UAS-mCherry, UAS-mCD8GFP]. hTau^421D^ (green) accumulates in discrete subcellular compartments. Right panels: hTau^421D^ accumulations labeled by arrowheads. **c** Left panel: hTau^421D^ (green) encapsulated in late endosomal compartments labeled with Rab7-GFP (red) in the soma of dHB9 expressing hTau neurons [*dHB9*-Gal4, UAS-hTau, UAS-Rab7-GFP, UAS-mCherry]. Middle panel: hTau^421D^ accumulations labeled by arrowheads. Right panel: Rab7-GFP (red), hTau^421D^ (green) and mCherry (blue). **d** Mander’s analyses of the spatial relationship of hTau^421D^ with endosomes labeled by either Rab4-GFP (*n* = 12), Rab5-GFP (*n* = 12) or Rab7-GFP (*n* = 11). M1 is the fraction of each Rab protein overlapping with Tau^421D^, while M2 is the fraction of Tau^421D^ signal overlapping with each Rab protein signal. **e**, **f** Left panels: hTau^421D^ accumulations (green) in the soma (**e**) and axons (**f**) of hHB9 neurons (mCherry, blue) expressing hTau together with retromer inhibition [*dHB9*-Gal4, UAS-hTau, UAS-Vps26^Ri^, UAS-mCherry]. Right panels: hTau^421D^ accumulations labeled by arrowheads. **g** Relative area of hTau^421D^ accumulations normalized to neuronal area in dHB9 neurons expressing hTau [*dHB9*-Gal4, UAS-hTau, UAS-mCherry, UAS-mCD8GFP] and when retromer is inhibited [*dHB9*-Gal4, UAS-hTau, UAS-mCherry, UAS-Vps26^Ri^]. Data are presented as mean values +/− SEM. **h** Intensity of hTau^421D^ accumulations normalized to mCherry in dHB9 neurons expressing hTau [*dHB9*-Gal4, UAS-hTau, UAS-mCherry, UAS-mCD8GFP] and when retromer is inhibited [*dHB9*-Gal4, UAS-hTau, UAS-mCherry, UAS-Vps26^Ri^]. Data are presented as mean values +/− SEM. **i** Left panel: hTau^421D^ (green) encapsulated in late endosomal compartments labeled with Rab7-GFP (red) in the soma of *dHB9* expressing hTau neurons when retromer is inhibited [*dHB9*-Gal4, UAS-hTau, UAS-Rab7-GFP, UAS-mCherry, UAS-Vps26^Ri^]. Middle panel: hTau^421D^ accumulations labeled by arrowheads. Right panel: Rab7-GFP (red), hTau^421D^ (green) and mCherry (blue). Unpaired two-tailed *t*-test, ***p < 0.001, hTau vs. hTau plus Vps26^Ri^ (*n* = 11 neurons examined over three independent experiments) (**g**). Unpaired two-tailed *t*-test, **p = 0.0039, hTau vs. hTau plus Vps26^Ri^ (*n* = 11 neurons examined over three independent experiments) (**h**). n indicates independent biological replicates. Source data are provided as a Source Data file.

As the increased production of hTau^421D^ was critical to the enhancement of hTau toxicity by retromer depletion, we next sought to investigate the localization of this species of Tau within neurons with single cell resolution. To do this, we labeled a small subset of glutamatergic neurons in the *Drosophila* nervous system characterized by the expression of the transcription factor dHB9^59,60^ having confirmed these neurons also express dTau (Supplementary Fig. 4a). Next, we expressed hTau in dHb9 neurons and examined the intracellular localization of full-length and truncated hTau. While full-length hTau was distributed throughout the neuron (Supplementary Fig. 4b), hTau^421D^ was localized to discrete compartments within both the neuron axon and soma (Fig. 6a, b). We assessed the nature of these compartments using markers specific for intracellular trafficking organelles. We observed poor localization with markers for many organelles including early endosomes labeled by Rab4 and Rab5 (Supplementary Fig. 4c, d). However, we did observe encapsulation of hTau^421D^ accumulations within compartments labeled by Rab7, a marker of late endosomes (Fig. 6c, Supplementary Movie 1). We quantified this spatial relationship using Manders analysis^61^ (Fig. 6d). We examined the overlap of hTau^421D^ with Rab4, Rab5 and Rab7 labeled compartments. We found no significant overlap of hTau^421D^ between Rab4 and Rab5 compartments. In contrast, the overlap between hTau^421D^ and Rab7 compartments was highly significant (*p* < 0.001). This indicated hTau^421D^ accumulations were largely within Rab7 labeled late endosomes. We also examined if hTau^421D^ accumulated in DC neurons (Supplementary Fig. 4e). Similar to HB9 neurons, when hTau was expressed in DC neurons, we also observed hTau^421D^ accumulations in Rab7 labeled compartments within their soma (Supplementary Fig. 4f). Interestingly, a recent report has also demonstrated colocalization of hTau^421D^ with Rab7 in postmortem brains of AD patients^62^.

We next examined the levels and localization of hTau^421D^ in neurons when retromer was depleted. We observed a dramatic increase in the immunohistochemical levels of hTau^421D^ (Fig. 6e, f), consistent with our protein blot measurements (Fig. 3e, f). We quantified this increase and found hTau^421D^ levels increased in area by 3.5-fold (*p* < 0.001) per neuron and in intensity by 2.5-fold (*p* < 0.01) per neuron when VPS26 was inhibited compared to neurons which express hTau alone (Fig. 6g, h). Like neurons expressing hTau alone, the increased accumulations of hTau^421D^ when retromer was inhibited were also restricted to Rab7 labeled late endosomes (Fig. 6i). Rab7 has previously been shown to interact directly with the retromer complex^40^, suggesting the possibility that hTau^421D^ might be accumulating in late endosomes due to a diminishment of this interaction. To test this hypothesis, we first confirmed using co-immunoprecipitation that *Drosophila* Rab7 could indeed interact with *Drosophila* VPS35 in vivo (Supplementary Fig. 5a). We also confirmed that this interaction did not occur with a ‘GDP-locked’ mutant version of Rab7 [Rab7^T22N^] (Supplementary Fig. 5a), sometimes known as ‘Dominant-Negative’ Rab7^63,64^.

**Fig. 7:**
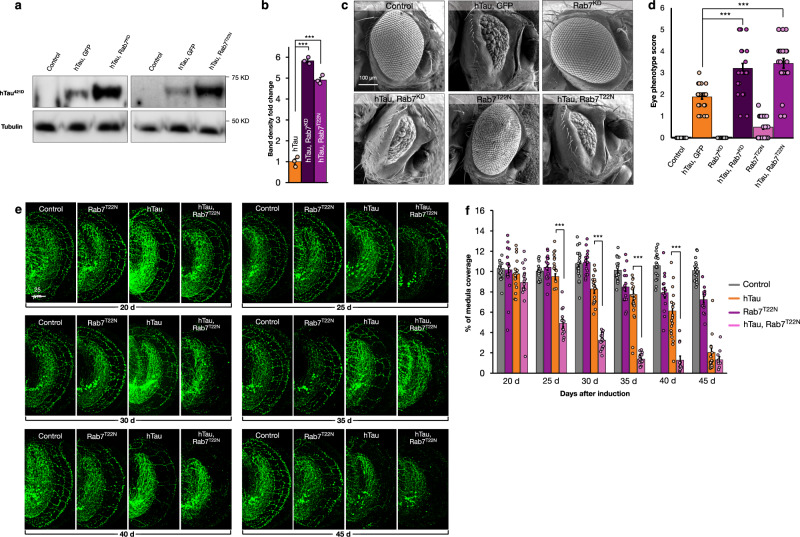
Rab7 inhibition increases Tau truncation. **a** hTau^421D^ protein level analyses from head lysates of controls [GMR-Gal4, UAS-mCD8GFP], hTau expressing [GMR-Gal4, UAS-hTau, UAS-DeGradFP] and hTau expressing together with Rab7 knock-down (Rab7^KD^) [GMR-Gal4, UAS-hTau, Rab7^EYFP/ΔRab7^, UAS-DeGradFP] or Rab7^T22N^ expression [GMR-Gal4, UAS-hTau, UAS-Rab7^T22N^] animals. **b** Quantification of hTau^421D^ protein levels from head lysates of controls [GMR-Gal4, UAS-mCD8GFP], hTau expressing [GMR-Gal4, UAS-hTau, UAS-DeGradFP] and hTau expressing together with Rab7 knock-down (Rab7^KD^) [GMR-Gal4, UAS-hTau, Rab7^EYFP/ΔRab7^, UAS-DeGradFP] or Rab7^T22N^ expression [GMR-Gal4, UAS-hTau, UAS-Rab7^T22N^] animals. Data are presented as mean values +/− SEM. **c** Eyes from control [GMR-Gal4, UAS-mCD8GFP], hTau expressing [GMR-Gal4, UAS-hTau, UAS-mCD8GFP], Rab7 knockdown alone (Rab7^KD^) [GMR-Gal4, Rab7^EYFP/ΔRab7^, UAS-DeGradFP], hTau expressing with Rab7^KD^ [GMR-Gal4, UAS-hTau, Rab7^EYFP/ΔRab7^, UAS-DeGradFP], Rab7 inhibition (Rab7^T22N^) alone [GMR-Gal4, UAS-Rab7^T22N^, UAS-mCD8GFP], and hTau expression together with Rab7^T22N^ [GMR-Gal4, UAS-hTau, UAS-Rab7^T22N^] animals. **d** Eye disruption scores from control [GMR-Gal4, UAS-mCD8GFP], hTau expressing [GMR-Gal4, UAS-hTau, UAS-DeGradFP], Rab7^KD^ [GMR-Gal4, Rab7^EYFP/ΔRab7^, UAS-DeGradFP], hTau expressing with Rab7^KD^ [GMR-Gal4, UAS-hTau, Rab7^EYFP/ΔRab7^, UAS-DeGradFP], Rab7^T22N^ [GMR-Gal4, UAS-Rab7^T22N^, UAS-mCD8GFP], and hTau expression together with Rab7^T22N^ [GMR-Gal4, UAS-hTau, UAS-Rab7^T22N^] animals. Data are presented as mean values +/− SEM. **e** Example DC neuron medulla axons from control [*ato*-Gal4, UAS-mCherry, UAS-mCD8GFP, *tubulin*-Gal80^ts^], Rab7^T22N^ expression alone [*ato*-Gal4, UAS-Rab7^T22N^, UAS-mCherry, *tubulin*-Gal80^ts^], hTau expression alone [*ato*-Gal4, UAS-hTau, UAS-mCherry, UAS-mCD8GFP, *tubulin*-Gal80^ts^], and hTau expression together with Rab7 inhibition [*ato*-Gal4, UAS-hTau, UAS-Rab7^T22N^, UAS-mCherry, *tubulin*-Gal80^ts^] animals from 20 to 45 days after onset of transgene expression. Gaps in axonal structure induced by hTau expression are indicated with arrowheads, when < 4% axons are present, the medulla is outlined with dashed lines. **f** Quantification of medulla area occupied by DC neuron from control [*ato*-Gal4, UAS-mCherry, UAS-mCD8GFP, *tubulin*-Gal80^ts^], Rab7^T22N^ expression alone [*ato*-Gal4, UAS-Rab7^T22N^, UAS-mCherry, *tubulin*-Gal80^ts^], hTau expression alone [*ato*-Gal4, UAS-hTau, UAS-mCherry, UAS-mCD8GFP, *tubulin*-Gal80^ts^], and hTau expression together with Rab7 inhibition [*ato*-Gal4, UAS-hTau, UAS-Rab7^T22N^, UAS-mCherry, *tubulin*-Gal80^ts^] 20–45 days after onset of transgene expression. Data are presented as mean values +/− SEM. Unpaired two-tailed *t*-test, *p* < 0.001, (**b**, hTau^421D^) for hTau (*n* = 3) vs. hTau plus Rab7^KD^ (*n* = 3), and *p* < 0.001 for hTau (*n* = 3) vs. hTau plus Rab7^T22N^ (*n* = 3). Kruskal–Wallis test, ***p < 0.001 for eye disruption scores across all genotypes with multiple comparisons between hTau (*n* = 26) and hTau plus Rab7^KD^ (*n* = 26) (Dunnett’s test ***p < 0.001), and hTau and hTau plus Rab7^T22N^ (*n* = 28). *n* = 32 for control, *n* = 30 for Rab7^KD^, and *n* = 28 for Rab7^T22N^ (**d**). Mann–Whitney test, *p* = 0.2928 for hTau (*n* = 15) vs. hTau plus Rab7^T22N^ (*n* = 18) (**f**, 20 days post induction, *n* = 15 for control and *n* = 16 for Rab7^T22N^). Mann–Whitney test, ***p < 0.001 for hTau (*n* = 16) vs. hTau plus Rab7^T22N^ (*n* = 15) (**f**, 25 days post induction, *n* = 18 for control and *n* = 17 for Rab7^T22N^). Mann–Whitney test, ***p < 0.001 for hTau (*n* = 20) vs. hTau plus Rab7^T22N^ (*n* = 17) (**f**, 30 days post induction, *n* = 20 for control and *n* = 15 for Rab7^T22N^). Mann–Whitney test, ***p < 0.001 for hTau (*n* = 20) vs. hTau plus Rab7^T22N^ (*n* = 18) (**f**, 35 days post induction, *n* = 17 for control and *n* = 15 for Rab7^T22N^). Mann–Whitney test, ***p < 0.001 for hTau (*n* = 20) vs. hTau plus Rab7^T22N^ (*n* = 12) (**f**, 40 days post induction, *n* = 18 for control and *n* = 14 for Rab7^T22N^). Mann–Whitney test, *p* = 0.7132 for hTau (*n* = 20) vs. hTau plus Rab7^T22N^ (*n* = 10) (**f**, 45 days post induction, *n* = 19 for control and *n* = 14 for Rab7^T22N^). *n* indicates independent biological replicates. Source data are provided as a Source Data file.

Having validated that retromer and Rab7 biochemically interact in *Drosophila*, we next wished to investigate if modulation of Rab7 activity could also alter hTau truncation. To deplete Rab7, we used deGradFP nanobodies^65^ to degrade Rab7 tagged in-frame with YFP inserted into the endogenous genetic locus^66^. We confirmed expression of deGradFP nanobodies throughout the brain could deplete Rab7 levels by 87% (*p* < 0.001) (Supplementary Fig. 5b, c). We next combined this method of Rab7 depletion with simultaneous expression of hTau. We found that depletion of Rab7 resulted in a ~6-fold (*p* < 0.001) increase in the levels of hTau^421D^ compared to hTau expression alone (Fig. 7a, b). To confirm this result, we also examined animals where GDP-locked Rab7^T22N^ was co-expressed with hTau. Similar to depletion of Rab7, expression of Rab7^T22N^ with hTau resulted in a ~5-fold (*p* < 0.001) increase of hTau^421D^ levels (Fig. 7a, b). In contrast, co-expression of either wildtype Rab7 or a ‘GTP-locked’ mutant Rab7 [Rab7^Q67L^]^63^ with hTau did not significantly alter truncated Tau levels (Supplementary Fig. 5d, e). We additionally confirmed that, similar to retromer inhibition, accumulation of hTau^421D^ when Rab7 was inhibited also required Dronc caspase activity (Supplementary Fig. 5f, g) and that levels of hTau^421D^ were not changed when hTau^421X^ was expressed together with Rab7 inhibition (Supplementary Fig. 5h). Therefore, inhibition of Rab7, like inhibition of retromer, results in increased production (and not clearance) of truncated Tau.

We then examined if phenotypes produced by hTau expression were altered when Rab7 activity was inhibited. Depletion of Rab7 using the nanobody approach had no effect on eye morphology (Fig. 7c, d). However, depletion of Rab7 together with hTau expression resulted in a 41.3% (*p* < 0.001) increase of the disruption of eye morphology compared to hTau expression alone. Expression of Rab7^T22N^ did modestly alter eye morphology when expressed alone (Fig. 7c, d), but co-expression with hTau also increased morphological disruption by 45.2% (*p* < 0.001) compared to hTau expression alone (Fig. 7c, d). In contrast, co-expression of Rab7^T22N^ together with cleavage-resistant hTau^D421E^ or truncated hTau^421X^ did not result in any additional disruption to eye morphology (Supplementary Fig. 6a, b, c). Expression of Rab7^WT^ or Rab7^Q67L^ also failed to additionally alter eye morphology when co-expressed with hTau (Supplementary Fig. 6d, e). Thus, inhibition of Rab7, similar to retromer, increases the hTau-dependent disruption of eye morphology consistent with a specific increase in the production of truncated Tau.

We then examined the effects of Rab7 inhibition in lifespan. Expression of Rab7^T22N^ with dTau-Gal4 modestly reduced lifespan (−8%, *p* < 0.001) (Supplementary Fig. 6f). However, expression of Rab7^T22N^ with hTau additionally reduced lifespan by −13.6% (*p* < 0.001) compared to hTau expression alone (Supplementary Fig. 6g).

Finally, we examined the effects of Rab7 inhibition on DC neuron degeneration. Expression of Rab7^T22N^ alone in DC neurons did not significantly affect medulla axons during early life though we did observe a modest but significant decline of axons after 35 days (Fig. 7e, f). In contrast, inhibition of Rab7 together with hTau resulted in a large increase in axonal loss compared to expression of hTau alone beginning from 25 days onwards (+48.3% at 25d, +60.8% at 30d, +82% at 35d, +79% at 40d [*p* < 0.001]). This acceleration was such that, after 35 days, almost all axons were lost in contrast to hTau expression alone (Fig. 7e, f). Therefore, in these assays, consistent with our observation of increased truncated Tau production, inhibition of Rab7, just as with depletion of retromer, increases the toxicity of human Tau expression. Our results support that the association of retromer with Rab7 is essential for the late endosomal trafficking of Tau and that disruption of either partner results in the accumulation of toxic truncated Tau species.

## Discussion

**Fig. 8:**
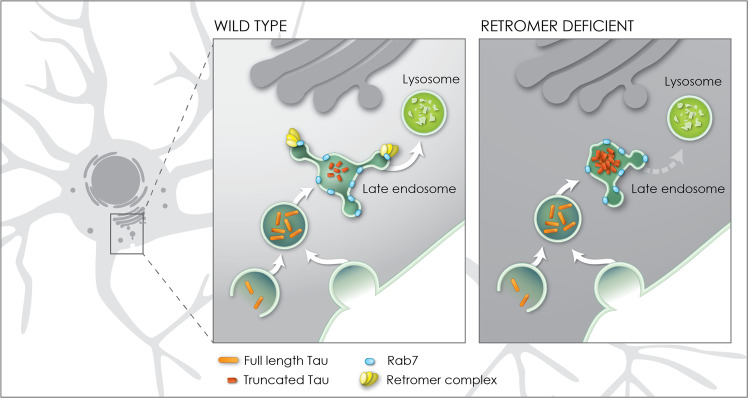
Model for enhancement of Tau toxicity and truncation by retromer depletion. In wildtype neurons full length Tau (orange) enters the endosomal network and is efficiently trafficked through the late endosome to the lysosome enabled by the interaction of the retromer complex (yellow) with the late endosome protein Rab7 (blue). When retromer or Rab7 activity is reduced, endosomal-lysosomal flux is retarded, exposing Tau to endosomal resident caspase activity which results in Tau truncation (red) and accumulation in the late endosome. Truncation of Tau enhances its neurotoxicity.

Deposition of abnormal, aggregated species of Tau protein is a characteristic feature of many neurodegenerative diseases^6^. In particular, Tau truncated at the C-terminus is both highly fibrillogenic and prone to aggregation^8,67–70^. One of these neurotoxic forms of Tau, Tau^421D^, is cleaved at Aspartate 421 by caspase activity^46,71–74^. Tau^421D^ truncation enhances the polymerization rate of Tau^43,68,70^ and this isoform is abundant in neurofibrillary tangles in both AD brains and those of other tauopathies^46,75,76^. Here, we show using models for human Tau toxicity in *Drosophila*, and recapitulated in mammalian neurons, that reduction of retromer activity amplifies the toxicity of human Tau expression upon both neuronal degeneration and animal viability in tandem with a specific and dramatic increase in the abundance of Tau^421D^. We show that this truncated form of Tau accumulates in neuronal late endosomes and establish that activity of the late endosome protein and retromer partner Rab7 is also essential to prevent accumulation of this truncated Tau species. Our data suggest a mechanism (Fig. 8) whereby depletion of retromer levels in neurons results in reduced retromer/Rab7 complexes. The endosomal trafficking of Tau, which can enter the network via endocytosis^77^ or autophagy^10,43,78,79^ en route to the lysosome^63^, is abnormally protracted by this depletion of retromer/Rab7 activity. This retardation of the endosomal flow of Tau extends exposure to caspases, such as human Caspases 3, 7 or 8^46^ or *Drosophila* Dronc^51,52^, active within the endosomal/lysosomal network^7^ resulting in abnormally enhanced levels of truncation. The resulting aberrant abundance of neurotoxic truncated Tau is detrimental to synapse and axon stability and ultimately animal viability.

Our evidence supports that the increased production of Tau^421D^ is singularly critical for and causal of the enhancement of Tau toxicity upon retromer deficiency. Firstly, while Tau truncation is dramatically increased when retromer levels are depleted, the levels of total Tau or several phosphorylated forms of Tau were unchanged, although potentially some additional Tau phosphoepitopes we did not examine could show alterations or perhaps some alterations were below our detection limit. Second, even though other modifications of Tau could occur^80^, inhibition of the caspase Dronc, which we show is necessary for cleavage of human Tau at Aspartate 421 in *Drosophila*, eliminates the enhancement of Tau toxicity by retromer depletion. Third, mutation of Aspartate 421 (hTau^D421E^) in human Tau also abrogates the amplification of Tau toxicity by retromer depletion. Finally, the toxicity of Tau already truncated at Aspartate 421 is not further increased by depletion of retromer activity. This latter result additionally establishes that retromer depletion increases the production of Tau^421D^ rather than perturbing the ability of neurons to eliminate this species. Similar results and specificity of Tau^421D^ production were established when Rab7 was depleted. Even in the absence of truncation, for example with hTau^D421E^ expression, elevated human Tau expression remains toxic to *Drosophila*, indicating this cleavage event is not solely responsible for all the detrimental effects of Tau in this model. But the results above in our models, together with the observed heightened toxicity of truncated Tau (e.g., hTau^421X^ expression), establish that Tau truncation is both necessary and sufficient to explain the intensification of Tau neurotoxicity in response to retromer depletion.

Interaction of the retromer complex with endosomal compartments, through either Rab5 or Rab7 proteins, is essential for the normal function of the endosomal-lysosomal pathway^81,82^. Trafficking in endosomal-lysosomal network is responsible for many key cellular processes including protein recycling and degradation, cellular signaling, and nutrient uptake^82^. Disruption of the endosomal-lysosomal system has been implicated in the pathology of several neurodegenerative diseases including AD^81^. The initial processing of Amyloid-beta precursor protein (APP), the precursor of Amyloid-beta plaques^83^, takes place within endosomes and pathological increases of amyloid-beta are dependent upon prolonged APP residence within endosomal compartments^81^. Consistent with a role for endosomal dysfunction in AD, endosomal compartments are enlarged in postmortem AD brains^84,85^, congruous with a retardation of endosomal-lysosomal flux^85^. Disease-specific lysosomal truncation of α-synuclein has been shown to occur in the brains of PD patients^86^ and Tau, like APP and α-synuclein, is also subject to processing and degradation via the endosomal-lysosomal system^79^. Mutations in the retromer component VPS35 are linked to familial Parkinson’s disease^17–22^ and cause an enlargement of endosomes^82^, consistent with a ‘traffic jam’ within the endosomal-lysosomal system^85^. In addition, the familial PD linked protein LRRK2^87,88^ has also been implicated in the aberrant processing of α-Synuclein through interaction with the Rab7 related protein PARK16/Rab7L1. Reduced retromer interaction with the endosomal network can also cause increased processing of APP into amyloid-beta^18^, though it is noteworthy that *Drosophila* do not produce Aβ in the absence of transgenic human BACE^89^, allowing us to exclude the effects we observe upon Tau toxicity with retromer depletion in our assays as a potentially indirect consequence of the modification of Aβ^29^ in our models. Consistent with our findings, recent studies have also shown that retromer-deficient cells have inhibited autophagic flux which can promote Tau aggregation^79^ and that Tau neuropathology is evident in mice harboring Parkinson’s disease-associated VPS35 mutations^90^. Our data support that hampered endosomal-lysosomal flux of Tau in retromer or Rab7 deficient neurons promotes the aberrant production of neurotoxic truncated species through prolonged exposure to endosomal resident caspases. Therefore, deficits in endosomal-lysosomal flux, a common feature in several neurodegenerative diseases, could contribute to protein processing dysfunction at multiple junctures.

The exploitation of *Drosophila* genetic models to study human neurodegenerative diseases has provided several insights into disease mechanisms^91^. Expression of disease-related proteins in the developing eye provides an easy-to-score and high-throughput assay to establish the relative toxicity of disease-associated moieties and to identify genetic or chemical modifiers^33,91,92^. Here we introduce a *Drosophila* model system that enables adult restricted expression of disease-associated proteins within a sparse and highly reproducible subset of CNS neurons. This assay enables the visualization and quantification of some of the earliest features of neurodegeneration, namely synapse loss and axonal retraction, with single neuron resolution within the context of an intact functioning brain and animal. This system enabled us to identify the differential effects of retromer depletion upon the earliest consequences to synapses and axons of Tau expression. Moreover, it enabled assays, such as assessment of the effect of inhibition of the caspase Dronc upon Tau truncation, and the consequent benefits to axon preservation, that would have been potentially obscured by the pleiotropic effects of broader manipulations. We envision this new model system, coupled with the automated tools we have developed to enable precise quantification of CNS synapse and axonal degeneration, will enable additional mechanistic insights into the intricate web of interactions between factors associated with susceptibility to human neurodegenerative disease.

## Methods

### Drosophila strains

UAS-hTau [2N4R] was described previously^32^. To generate UAS-hTau-421X, a stop codon was inserted to replace Serine 422 in hTau using KLD Enzyme Mix (New England Biolabs) and transgenes generated using pBID^93^ *Drosophila* expression vector. To inhibit retromer components we used UAS-RNAi-Vps35^94^ (BDSC# 38944), UAS-RNAi-Vps29^94^ (BDSC# 38963) and UAS-RNAi-Vps26^94^ (BDSC# 38937). For protein interactions with retromer we used UAS-Vps35HA^95^ and UAS-Vps29-HA^96^. To manipulate Rab7 we used UAS-Rab7-GFP (BDSC# 42705 and 42706), UAS-YFP-Rab7^[T22N]^^97^ (BDSC# 23235), UAS-Venus-Rab7^[WT]^^98^ and UAS-Venus-Rab7^[T22N]^^98^. For eye expression we employed GMR-Gal4^99^, for DC neuron expression *ato*-Gal4[14a]^38^, and for whole brain expression Elav-Gal4^100^ (BDSC# 458). Additional strains and procedures are described in supplemental methods.

### Drosophila phenotypic analysis

Eye phenotypes were imaged, using Leica DFC 3000 G and scored blind to genotype using a modified version of published criteria^32^ as described in Supplementary Fig. 1 and in supplementary methods. Preparation of scanning electron micrographs are described in supplemental methods and were imaged using a Zeiss Merlin Scanning Electron Microscope. To label DC neuron axons and express transgenes we generated a line that combined *ato*-Gal4^38^ with UAS-mCherry [6X]^101^. The expression of transgenes by this line during development was inhibited by combining it with temperature-sensitive Gal80 (Tub-Gal80^ts^)^34^ which suppresses Gal4-dependent expression at 18 °C but allows expression at 29 °C. Animals of the desired genotype were grown at 18 °C until 3 days after eclosion and then transferred to 29 °C. Under these expression conditions, control animals lived to a maximal age of 50 days. Entire adult brains were stained and imaged using a Zeiss LSM700. Quantification of DC neuron medulla axon coverage was performed blind to genotype using the threshold tool in ImageJ^102^. Briefly, medulla areas were cropped from Z projections of brain images. Pixel intensity was then set such that axons were above threshold. The percentage of medulla area occupied by axons was then measured. Brain dissection, staining procedures, and antibodies employed are described in supplementary methods.

### Protein measurements

Protein measurements were performed using adult head lysates and imaged using an Amersham Imager 680 or Vilber Lourmat Fusion FX. Densitometric analyses of western blots were quantitated using the Gels tool of ImageJ. The following antibodies were used: mouse anti-cleaved-Tau (Asp421) clone C3 (Millipore; 36-017) 1:1000, rabbit anti-TauC (1:20,000), rabbit anti-PHF-1 (Sigma) 1:2000, monoclonal anti-phospho-Tau (Ser202, Thr205) (AT8) (Thermo Fisher) 1:2000, monoclonal anti-phospho-Tau (Thr212, Ser214) (AT100) (Thermo Fisher) 1:2000, monoclonal anti-Spectrin (DSHB) 1:1000, monoclonal anti-alpha tubulin (Sigma) 1:2000, mouse anti-HA (Covance) 1:1000, guinea pig anti-Vps26 (gift from Hugo Bellen) (1:4000), HRP anti-mouse (Jackson Immunoresearch) (1:20,000), HRP anti-rabbit (Jackson ImmunoResearch) (1:20,000), HRP anti-guinea pig (1:20,000). Additional details on head lysate preparation, immunoprecipitation, and antibody staining procedures are described in supplemental methods.

### Mammalian cell culture

Immortalized CN1.4 mouse cortical neurons that stably express inducible forms of hTau [2N4R] were described previously^50^. To inhibit retromer in these lines we used lentivirus expression of murine VPS26A, VPS29 or VPS35 shRNA or a scrambled shRNA control. Cell culture conditions, lentivirus preparation, transduction, and protein measurement procedures are described in supplemental methods.

### Statistics

Prior to performing statistical analyses, the distribution of data was checked, for all datasets, using a Shapiro-Wilk normality test. In the event of non-normal distribution, even for one group in a large dataset (for example, a timepoint in axon degeneration dataset), a non-parametric test was chosen. The following tests were employed—for *Drosophila* eye phenotypes: non-parametric Mann–Whitney *U* test or Kruskal–Wallis test with Dunnett’s multiple comparison test; for synapse and axon degeneration: Unpaired two-tailed *t*-test, one-way ANOVA or Kruskal–Wallis test along with Dunnett’s multiple comparison test; for protein levels analysis: Unpaired two-tailed *t*-test or one-way ANOVA along with Tukey’s multiple comparison test. For multiple comparisons, adjusted *p* values are reported. Statistical analyses were performed using GraphPad PRISM 7.0a.

### Ethical approval

Studies using *Drosophila melanogaster* are not subject to institutional ethical approval however all experiments complied with relevant guidelines.

### Reporting summary

Further information on research design is available in the Nature Research Reporting Summary linked to this article.

## Supplementary information

Supplementary Information

Supplementary Movie 1

Reporting Summary

## Data Availability

Source data are provided with this paper. ﻿ Imaging data generated in this study have been deposited in the Zenodo database under accession code 10.5281/zenodo.6926423^103^. Unprocessed protein measurements and all other data generated in this study are provided in the Source Data file. All reagents including *Drosophila* strains and viral vectors are available upon request. Source data are provided with this paper.

## Source data

Source Data File

## Code availability

Custom Macros developed for this study are available at 10.5281/zenodo.5206099 and 10.5281/zenodo.5206253.
