# Supplementary material for: Retromer deficiency in Tauopathy models enhances the truncation and toxicity of Tau
Source: Nat Commun. 2022 Aug 27;13:5049. doi: 10.1038/s41467-022-32683-5 (PMC9420134; doi:10.1038/s41467-022-32683-5)
Supplement: Supplementary file 1 — Supplementary Information [file 41467_2022_32683_MOESM1_ESM.pdf]

Supplementary Information

Supplementary Fig. 1 Eye scoring criteria, dTau-Gal4 characterisation, stages of axon degeneration, assays of neuronal loss plus axon degeneration in context of broad hTau expression.

a Eye disruption scoring system (see methods). **b** Quantitative measurements of *Drosophila* Tau (*dTau*) mRNA levels in Control [W^{118}], *dTau*-Gal4 heterozygotes, and dTau mutant (*dTau*^{KO}) animals. Data are presented as mean values +/- SEM. **c** Examples of axon degeneration in hTau expressing [*ato*-Gal4, UAS-hTau, UAS-mCherry, UAS-mCD8GFP, *tubulin*-Gal80^{ts}] DC neurons 35 days after induction of transgene expression: (1) an axon with normal thickness, (2) a thinning axon and (3) a fragmenting axon. **d** DC neuron soma in control [*ato*-Gal4, UAS-His-GFP, UAS-mCherry, *tubulin*-Gal80^{ts}] and hTau expressing [*ato*-Gal4, UAS-hTau, UAS-His-GFP, UAS-mCherry, *tubulin*-Gal80^{ts}] animals from 25 to 45 days after onset of expression of hTau. **e** A magnified view of DC neurons' nuclei labeled with a GFP-tagged histone [*ato*-Gal4, UAS-His-GFP, UAS-mCherry, *tubulin*-Gal80^{ts}]. **f** Quantification of number of DC neuron soma for control [*ato*-Gal4, UAS-His-GFP, UAS-mCherry, *tubulin*-Gal80^{ts}] and hTau expressing [*ato*-Gal4, UAS-hTau, UAS-His-GFP, UAS-mCherry, *tubulin*-Gal80^{ts}] animals. Data are presented as mean values +/- SEM. **g** DC neuron axons in animals that express hTau under *dTau*-Gal4 control [*dTau*-Gal4, UAS-hTau, *ato*-LexA, LexAop-mCherry, *tubulin*-Gal80^{ts}] and controls [*dTau*-Gal4, UAS-mCD8GFP, *ato*-LexA, LexAop-mCherry, *tubulin*-Gal80^{ts}]. One-Way ANOVA, ***p<0.001, n = 9 samples examined over 3 independent experiments for all genotypes, with multiple comparisons between control and *dTau*-Gal4 (Tukey's test p<0.001), control and *dTau* KO (Tukey's test p<0.001), and *dTau*-Gal4 and *dTau*^{KO} (Tukey's test p<0.001) (**b**). One-Way ANOVA, p=0.8281, with multiple comparisons between control (n = 18) and hTau (n = 20) (Dunnett's test p=0.9606), control and hTau plus Vps35^{Ri} (n = 18) (Dunnett's test p=0.8744), control and hTau plus Vps29^{Ri} (n = 20) (Dunnett's test p=0.574), and control and hTau plus Vps26^{Ri} (n = 16) (Dunnett's test p=0.9477) (**f**, 25 days post induction). One-Way ANOVA, p=0.1735, with multiple comparisons between control (n = 20) and hTau (n = 20) (Dunnett's test p=0.2469), control and hTau plus Vps35^{Ri} (n = 20) (Dunnett's test p=0.9962), control and hTau plus Vps29^{Ri} (n = 20) (Dunnett's test p=0.9523), and control and hTau plus Vps26^{Ri} (n = 19) (Dunnett's test p=0.9629) (**f**, 30 days post induction). One-Way ANOVA, p=0.6276 with multiple comparisons between control (n = 17) and hTau (n = 20) (Dunnett's test p=0.998), control and hTau plus Vps35^{Ri} (n = 18) (Dunnett's test p=0.5542), control and hTau plus Vps29^{Ri} (n = 20) (Dunnett's test p=0.7446), and control and hTau plus Vps26^{Ri} (n = 20) (Dunnett's test p=0.5862) (**f**, 35 days post induction). One-Way ANOVA, p=0.714 with multiple comparisons between control (n = 18) and hTau (n = 19) (Dunnett's test p=0.9863), control and hTau plus Vps35^{Ri} (n = 19) (Dunnett's test p=0.7225), control and hTau plus Vps29^{Ri} (n = 20) (Dunnett's test p=0.8422), and control and hTau plus Vps26^{Ri} (n = 20) (Dunnett's test p=0.4904) (**f**, 40 days post induction). One-Way ANOVA, p=0.7373 with multiple comparisons between control (n = 19) and hTau (n = 20) (Dunnett's test p=0.9961), control and hTau plus Vps35^{Ri} (n = 12) (Dunnett's test p=0.7197), control and hTau plus Vps29^{Ri} (n = 13) (Dunnett's test p=0.9617), and control and hTau plus Vps26^{Ri} (n = 15) (Dunnett's test p=0.9952) (**f**, 45 days post induction). n indicates independent biological replicates. Source data are provided as a Source Data file.

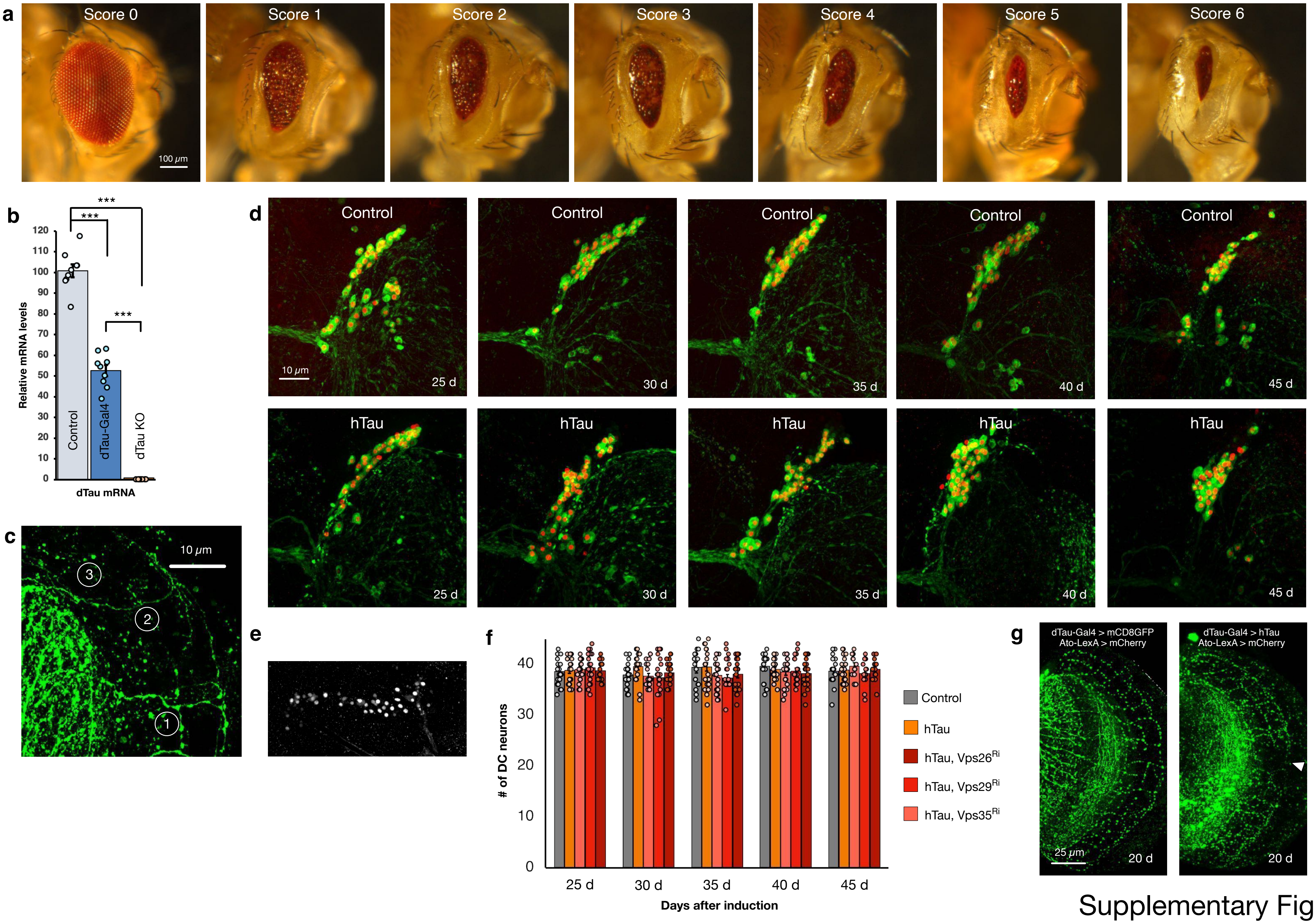

Supplementary Fig. 1

Supplementary Fig. 2 ***Drosophila*** **retromer RNAi quantification, eye phenotypes, adult lifespan and axon degeneration plus human retromer transgene axon degeneration.**

a Protein level analyses of head lysates of animals expressing transgenic RNAi lines against retromer components. To evaluate Vps35, Vps29 RNAi lines, we co-expressed a HA-tagged transgene of either protein together with the RNAi construct and probed with an anti-HA antibody [control: Elav-Gal4, UAS-Vps35-HA, UAS-mCD8GFP and RNAi: Elav-Gal4, UAS-Vps35-HA, UAS-Vps35^{Ri}, control: Elav-Gal4, UAS-Vps29-HA, UAS-mCD8GFP and RNAi: Elav-Gal4, UAS-Vps29-HA, UAS-Vps29^{Ri}]. For Vps26, we expressed Vps26^{Ri} [control: Elav-Gal4, UAS-mCD8GFP and RNAi: Elav-Gal4, UAS-Vps26^{Ri}] and measured endogenous VPS26 levels. **b** Quantification of VPS35-HA in control [Elav-Gal4, UAS-Vps35-HA, UAS-mCD8GFP] and RNAi [Elav-Gal4, UAS-Vps35-HA, UAS-Vps35^{R}] animals, and VPS29-HA in control [Elav-Gal4, UAS-Vps29-HA, UAS-mCD8GFP] and RNAi [Elav-Gal4, UAS-Vps29-HA, UAS-Vps29^{Ri}] animals, and endogenous VPS26 protein levels in control [Elav-Gal4, UAS-mCD8GFP] and RNAi [Elav-Gal4, UAS-Vps26^{Ri}]. Data are presented as mean values +/- SEM. **c** Inhibition of retromer components in *Drosophila* eyes does not cause disruption in [GMR-Gal4, UAS-Vps35^{Ri}], or [GMR-Gal4, UAS-Vps29^{Ri}], or [GMR-Gal4, UAS-Vps26^{Ri}] animals. **d** Survival analyses of control [*dTau*-Gal4, UAS-Cherry^{Ri}, *tubulin*-Gal80^{ts}] animals and knockdown of VPS35 [*dTau*-Gal4, UAS-Vps35^{Ri}, *tubulin*-Gal80^{ts}], VPS29 [*dTau*-Gal4, UAS-Vps29^{Ri}, *tubulin*-Gal80^{ts}], and VPS26 [*dTau*-Gal4, UAS-Vps26^{Ri}, *tubulin*-Gal80^{ts}]. **e** Quantification of percentage of medulla covered by axons when VPS35 alone [*ato*-Gal4, UAS-Vps35^{Ri}, UAS-mCherry, UAS-mCD8GFP, *tubulin*-Gal80^{ts}], or VPS29 alone [*ato*-Gal4, UAS-Vps29^{Ri}, UAS-mCherry, UAS-mCD8GFP, *tubulin*-Gal80^{ts}], or VPS26 alone [*ato*-Gal4, UAS-Vps26^{Ri}, UAS-mCherry, UAS-mCD8GFP, *tubulin*-Gal80^{ts}] were depleted in adult Atonal neurons. Data are presented as mean values +/- SEM. **f** Example DC neuron medulla axons from control [*ato*-Gal4, UAS-mCherry, UAS-mCD8GFP, *tubulin*-Gal80^{ts}], VPS26-V5 overexpressing [*ato*-Gal4, UAS-mCherry, UAS-Vps26-V5, *tubulin*-Gal80^{ts}], VPS29-HA overexpressing [*ato*-Gal4, UAS-mCherry, UAS-Vps29-HA, *tubulin*-Gal80^{ts}], and VPS35-HA overexpressing [*ato*-Gal4, UAS-mCherry, UAS-Vps35-HA, *tubulin*-Gal80^{ts}] animals, 30 days after onset of transgene expression. Overproduction of any retromer component in neurons induces axonal retraction not found in controls. Unpaired two-tailed *t*-test, *p=0.0288 for control (n = 3) vs. Vps35^{Ri} (n = 3) (**b**, anti-HA), Unpaired two-tailed *t*-test, ***p<0.001 for control (n = 3) vs. Vps29^{Ri} (n = 3) (**b**, anti-HA), Unpaired two-tailed *t*-test, ***p<0.001 for control (n = 3) vs. Vps26^{Ri} (n = 3) (**b**, anti-VPS26). Mantel-Cox test, ***p<0.001 for survival analysis of control (n = 198) vs. Vps35^{Ri} (n = 199) (**d**), Mantel-Cox test, p=0.8104 for survival analysis of control vs. Vps29^{Ri} (n = 188) (**d**), Mantel-Cox test, ***p<0.001 for survival analysis of control vs. Vps29^{Ri} (n = 185) (**d**). Kruskal-Wallis test, p=0.6684 (**e**, 25 days post induction) with multiple comparisons between control (n = 18) and Vps35^{Ri} (n = 15) (Dunnett's test p=0.8996), control and Vps29^{Ri} (n = 20) (Dunnett's test p=0.7964), and control and Vps26^{Ri} (n = 14) (Dunnett's test p=0.8237), Kruskal-Wallis test, p=0.201 (**e**, 30 days post induction) with multiple comparisons between control (n = 20) and Vps35^{Ri} (n = 11) (Dunnett's test p=0.195), control and Vps29^{Ri} (n = 16) (Dunnett's test p=0.5146), control and Vps26^{Ri} (n = 13) (Dunnett's test p=0.1235), Kruskal-Wallis test, p=0.7499 (**e**, 35 days post induction) with multiple comparisons between control (n = 17) and Vps35^{Ri} (n = 15) (Dunnett's test p=0.8498), control and Vps29^{Ri} (n = 13) (Dunnett's test p=0.7179), and control and Vps26^{Ri} (n = 12) (Dunnett's test p=0.7624), Kruskal-Wallis test, p=0.3242 (**e**, 40 days post induction) with multiple comparisons between control (n = 18) and Vps35^{Ri} (n = 12) (Dunnett's test p=0.5644), control and Vps29^{Ri} (n = 14) (Dunnett's test p=0.6892), and control and Vps26^{Ri} (n = 13) (Dunnett's test p=0.1486), Kruskal-Wallis test, p=0.1896 (**e**, 45 days post induction) with multiple comparisons between control (n = 19) and Vps35^{Ri} (n = 11) (Dunnett's test p=0.9188), control and Vps29^{Ri} (n = 12) (Dunnett's test p=0.1819), and control and Vps26^{Ri} (n = 11) (Dunnett's test p=0.9373). n indicates independent biological replicates. Source data are provided as a Source Data file.

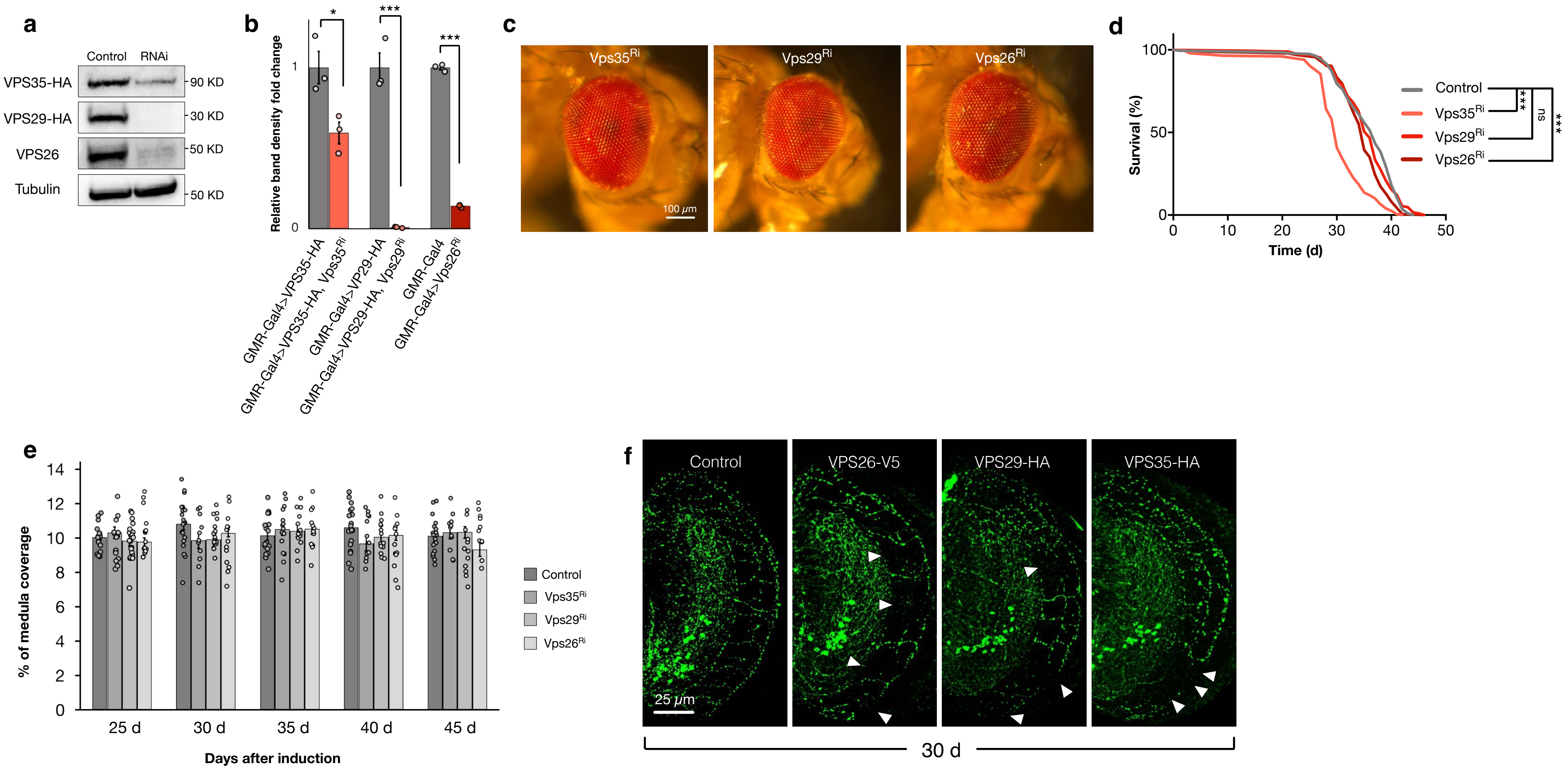

Supplementary Fig. 2

Supplementary Fig. 3 Levels of truncated Tau in *Drosophila* and mammalian cell lines with retromer inhibition or caspase inhibition plus effects of caspase inhibition upon adult lifespan.

a hTau^{421D} protein levels analyses of head lysates when retromer activity is restored via expression of an RNAi-resistant human homologue in hTau expressing animals when VPS35 [GMR-Gal4, UAS-hTau, UAS-Vps35^{Ri}, UAS-hVPS35], VPS29 [GMR-Gal4, UAS-hTau, UAS-Vps29^{Ri}, UAS-hVPS29], or VPS26 [GMR-Gal4, UAS-hTau, UAS-Vps26^{Ri}, UAS-hVPS26A] was inhibited. **b** Quantification of hTau^{421D} levels in hTau expressing animals [GMR-Gal4, UAS-hTau] when in VPS35 inhibition plus rescue [UAS-Vps35^{Ri}, UAS-hVPS35], or VPS29 inhibition plus rescue [UAS-Vps29^{Ri}, UAS-hVPS29], or VPS26 inhibition plus rescue [UAS-Vps26^{Ri}, UAS-hVPS26A]. Data are presented as mean values +/- SEM. **c** Tau induction, and consequently Tau truncation at Asp 421, in CN1.4 cells when a gradient concentration of doxycycline is applied. **d** Quantitative measurements of murine VPS26A, VPS29, and VPS35 mRNA levels in CN1.4 cells upon infection with the corresponding shRNA. Data are presented as mean values +/- SEM. **e** Protein analyses of head lysates in control [GMR-Gal4, UAS-mCD8GFP], hTau expressing [GMR-Gal4, UAS-hTau, UAS-Control^{Ri}], and hTau expression together with caspase depletion [GMR-Gal4, UAS-hTau, UAS-Drice^{Ri}, or UAS-Dredd^{Ri}, or UAS-Dronc^{Ri}] animals, probed with anti-Tau^{421D} antibody with short and long exposure times. **f** Quantification of Tau^{421D} levels in control [GMR-Gal4], hTau expressing [GMR-Gal4, UAS-hTau, UAS-Control^{Ri}], and hTau expression together with caspase depletion [GMR-Gal4, UAS-hTau, UAS-Drice^{Ri}, or UAS-Dredd^{Ri}, or UAS-Dronc^{Ri}] animals. Data are presented as mean values +/- SEM. **g** Analyses of hTau^{421D} protein levels in head lysates of control animals [GMR-Gal4, UAS-mCD8GFP], hTau expression alone [GMR-Gal4, UAS-mCD8GFP, UAS-hTau, UAS-Control^{Ri}], hTau expression together with RNAi inhibition of Vps26 [GMR-Gal4, UAS-mCD8GFP, UAS-hTau, UAS-Vps26^{Ri}], and hTau expressing individuals with concomitant retromer and Dronc inhibition [GMR-Gal4, UAS-hTau, UAS-Vps26^{Ri}, UAS-Dronc^{Ri}]. **h** Quantification of hTau^{421D} levels in control [GMR-Gal4, UAS-mCD8GFP], hTau expressing [GMR-Gal4, UAS-mCD8GFP, UAS-hTau, UAS-Control^{Ri}], hTau expression together with RNAi inhibition of Vps26 [GMR-Gal4, UAS-mCD8GFP, UAS-hTau, UAS-Vps26^{Ri}], and hTau expressing together with concomitant retromer and Dronc inhibition [GMR-Gal4, UAS-hTau, UAS-Vps26^{Ri}, UAS-Dronc^{Ri}] animals. Data are presented as mean values +/- SEM. **i** Analyses of hTau^{421D} protein levels in head lysates of control animals [GMR-Gal4, UAS-mCD8GFP], hTau expression alone [GMR-Gal4, UAS-hTau, UAS-mCD8GFP], and hTau expressing individuals with concomitant Dronc overexpression [GMR-Gal4, UAS-hTau, UAS-Dronc-GFP]. **j** Quantification of of hTau^{421D} protein levels in head lysates of control animals [GMR-Gal4, UAS-mCD8GFP], hTau expression alone [GMR-Gal4, UAS-hTau, UAS-mCD8GFP], and hTau expressing individuals with concomitant Dronc overexpression [GMR-Gal4, UAS-hTau, UAS-Dronc-GFP]. Data are presented as mean values +/- SEM. **k** Survival analyses in Dronc^{Ri} expression alone [*dTau*-Gal4, UAS-mCD8GFP, UAS-Dronc^{Ri}, *tubulin*-Gal80^{ts}] versus control animals [*dTau*-Gal4, UAS-mCD8GFP, UAS-Cherry^{Ri}, *tubulin*-Gal80^{ts}]). **l** Survival analyses of hTau expressing [*dTau*-Gal4, UAS-hTau, UAS-Cherry^{Ri}, *tubulin*-Gal80^{ts}] adults and hTau expression together with Dronc depletion [*dTau*-Gal4, UAS-hTau, UAS-Dronc^{Ri}, *tubulin*-Gal80^{ts}]. Unpaired two-tailed *t*-test, $^{**}p=0.009$ for hTau plus Vps35^{Ri} (n = 3) vs. hTau plus Vps35^{Ri} plus hVPS35 (n = 3), Unpaired two-tailed *t*-test, $^{**}p=0.0059$ for hTau plus Vps29^{Ri} (n = 3) vs. hTau plus Vps29^{Ri} plus hVPS29 (n = 3). Unpaired two-tailed *t*-test, $^{**}p=0.0059$ for hTau plus Vps29^{Ri} (n = 3) vs. hTau plus Vps29^{Ri} plus hVPS29 (n = 3). Unpaired two-tailed *t*-test, $^{**}p=0.0044$ for hTau plus Vps26^{Ri} (n = 3) vs. hTau plus Vps26^{Ri} plus hVPS26 (n = 3). Unpaired two-tailed *t*-test, $^{**}p=0.0018$ for hTau plus shRNA-scrambled (Scr) (n = 9 samples examined over 3 independent experiments) vs. hTau plus shRNA-VPS35 (n = 9 samples examined over 3 independent experiments), Unpaired two-tailed *t*-test, $^{***}p=0.0006$ for hTau plus shRNA-scrambled (Scr) vs. hTau plus shRNA-VPS29 (n = 9 samples examined over 3 independent experiments), Unpaired two-tailed *t*-test, $^{***}p=0.0008$ for hTau plus shRNA-scrambled (Scr) vs. hTau plus shRNA-VPS26 (n = 9 samples examined over 3 independent experiments). One-Way ANOVA, $^{***}p<0.001$, with multiple comparisons between hTau (n = 3) and hTau plus Drice^{Ri} (n = 3) (Dunnett's test $^{***}p<0.001$), hTau and hTau plus Dredd^{Ri} (n = 3) (Dunnett's test $^{***}p<0.001$), and hTau and hTau plus Dronc^{Ri} (Dunnett's test $^{**}p=0.0071$) (**f**). Unpaired two-tailed *t*-test, $^{**}p=0.0015$ for hTau plus Vps26^{Ri} (n = 3) vs. hTau plus Vps26^{Ri} plus Dronc^{Ri} (n = 3) (**h**, n = 3 for hTau). Unpaired two-tailed *t*-test, $^{***}p<0.001$ for hTau (n = 3) vs. hTau plus Dronc-GFP (n = 3) (**j**). Mantel-Cox test, $^{***}p<0.001$ for survival analysis of control (n = 202) vs. Dronc^{Ri} (n = 167), and $^{***}p<0.001$ for hTau (n = 148) vs. hTau plus Dronc^{Ri} (n = 155). n indicates independent biological replicates. Source data are provided as a Source Data file.

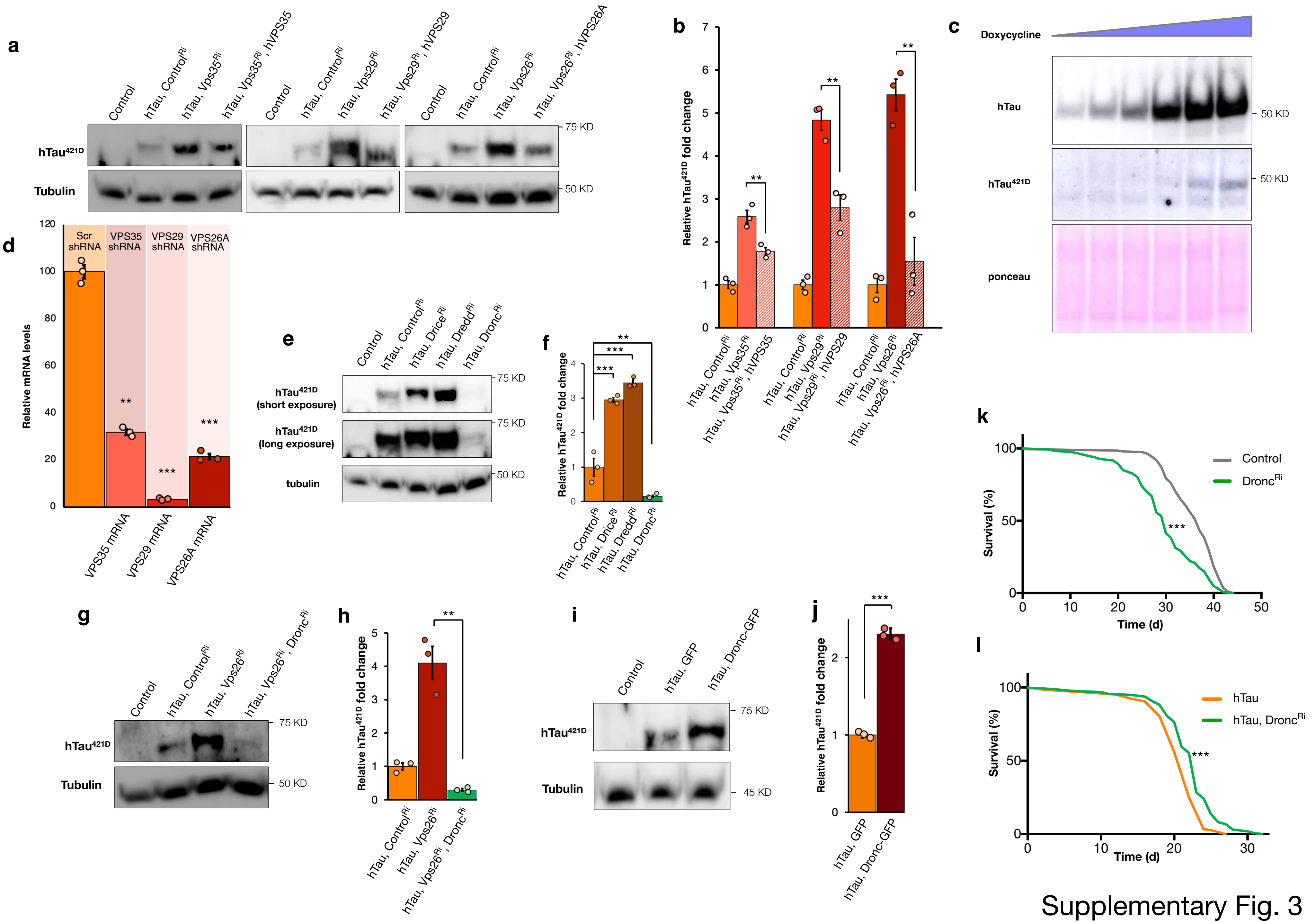

Supplementary Fig. 3

Supplementary Fig. 4 Overlap of *dHB9* expression with *dTau* expression, comparison of localisation full length and truncated Tau in subcellular compartments plus truncated Tau localisation with broad Tau expression.

a Intersection of *dHB9*-QF2 and *dTau*-Gal4 drivers [*dHB9*-QF2, QUAS-His2B-Cherry, *dTau*-Gal4, UAS-His2A-GFP] in *Drosophila* larval brains. **b** Distribution of full-length hTau (green) in *dHB9* neurons (blue) [*dHB9*-Gal4, UAS-hTau, UAS-mCherry]. **c** $hTau^{421D}$ (green; solid arrowheads) in neurons with intracellular labelling of Rab4 (open arrowheads) [*dHB9*-Gal4, UAS-hTau, UAS-Rab4-GFP, UAS-mCherry], or **d** Rab5 (open arrowhead) [*dHB9*-Gal4, UAS-hTau, UAS-Rab5-GFP, UAS-mCherry] compartments labelled in red. **e** DC neuron (innermost inset, continuous line) within a DC cluster (outermost inset, broken line) in an animal expressing hTau [*ato*-Gal4, UAS-hTau, UAS-Rab7-GFP, UAS-mCherry, *tubulin*-$Gal80^{ts}$]. **f** $hTau^{421D}$ (green) accumulation in Rab7 (red) labeled compartments in the soma of the DC neuron highlighted in e.

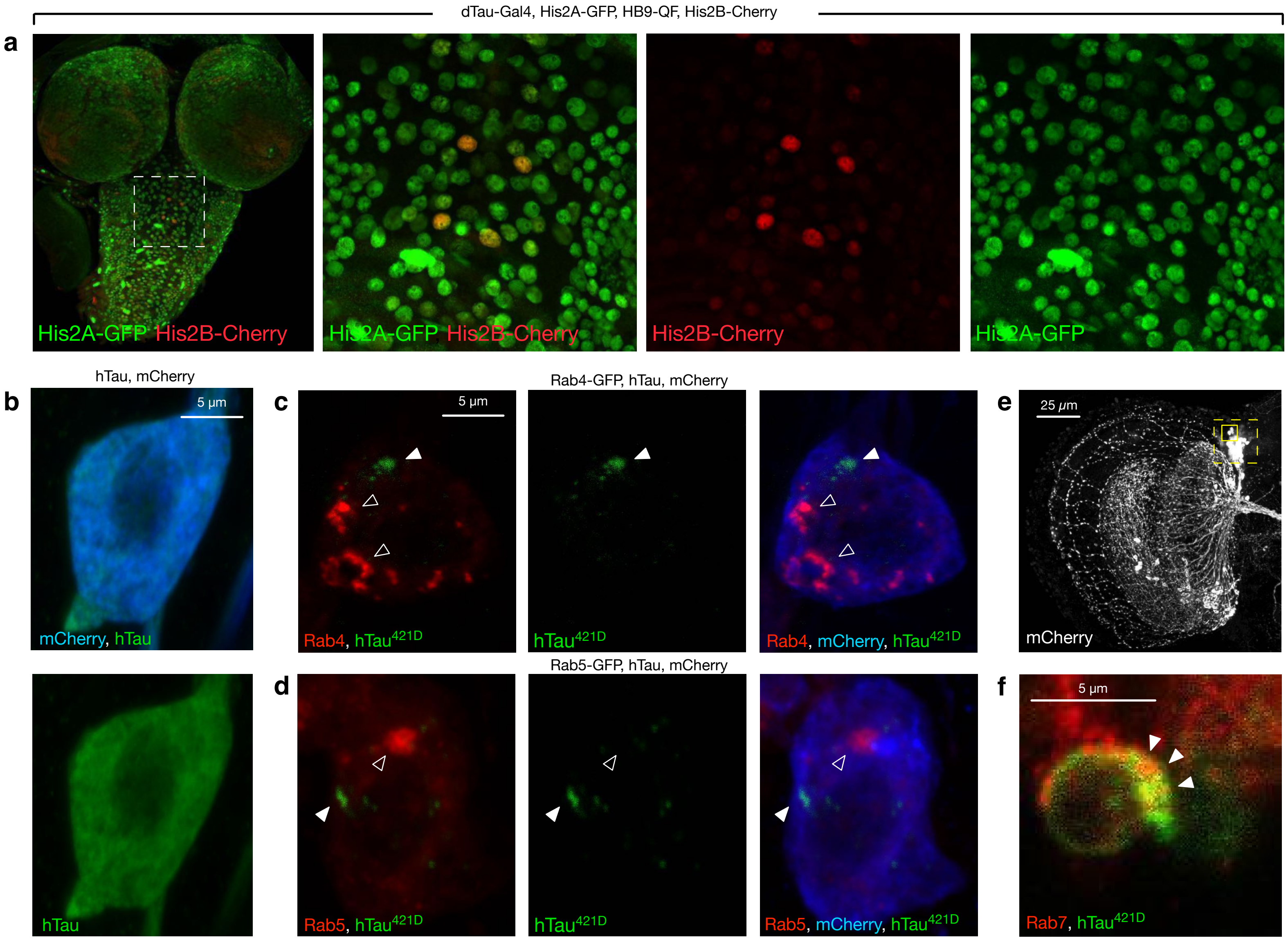

Supplementary Fig. 4

Supplementary Fig. 5 Co-immunoprecipitation of VPS35 and Rab7, Quantification of Rab7 inhibition, plus levels of truncated Tau and hTau^{421X} when Rab7 is inhibited.

a Co-IP from head lysates of GMR-Gal4, UAS-Vps35-HA, UAS-Rab7^{WT}-GFP (top panels) or GMR-Gal4, UAS-Vps35-HA, UAS-Rab7^{T22N}-GFP (bottom panels) animals. VPS35-HA was pulled down using an anti-HA antibody. Rab7-GFP was then probed using anti-GFP antibody. **b** Rab7 protein level analyses in head lysates from Rab7^{EYFP} control [GMR-Gal4, Rab7$^{EYFP/+}$] and Rab7^{EYFP} together with DeGradFP expression [GMR-Gal4, Rab7$^{EYFP/+}$, UAS-DeGradFP] animals. **c** Quantification of Rab7^{EYFP} levels in control [GMR-Gal4, Rab7$^{EYFP/+}$] and Rab7^{EYFP} together with DeGradFP expression [GMR-Gal4, Rab7$^{EYFP/+}$, UAS-DeGradFP] animals. Data are presented as mean values +/- SEM. **d** hTau^{421D} protein analyses in head lysates from control [GMR-Gal4, UAS-mCD8GFP], hTau expressing [GMR-Gal4, UAS-hTau, UAS-mCD8GFP], hTau and Rab7^{WT} expressing [GMR-Gal4, UAS-hTau, UAS-Rab7^{WT}-GFP], hTau and Rab7^{Q67L} expressing [GMR-Gal4, UAS-hTau, UAS-Rab7^{Q67L}-GFP], and hTau and Rab7^{T22N} expressing [GMR-Gal4, UAS-hTau, UAS-Rab7^{T22N}-GFP] animals. **e** Quantification of hTau^{421D} levels in control [GMR-Gal4, UAS-mCD8GFP], hTau expressing [GMR-Gal4, UAS-hTau, UAS-mCD8GFP], hTau and Rab7^{WT} expressing [GMR-Gal4, UAS-hTau, UAS-Rab7^{WT}-GFP], hTau and Rab7^{Q67L} expressing [GMR-Gal4, UAS-hTau, UAS-Rab7^{Q67L}-GFP], and hTau and Rab7^{T22N} expressing [GMR-Gal4, UAS-hTau, UAS-Rab7^{T22N}-GFP] animals. Data are presented as mean values +/- SEM. **f** Protein analyses of head lysates from control [GMR-Gal4, UAS-mCD8GFP], hTau expressing [GMR-Gal4, UAS-hTau, UAS-mCD8GFP, UAS-Control^{Ri}], and hTau expression together with Rab7^{T22N} [GMR-Gal4, UAS-hTau, UAS-Rab7^{T22N}-GFP, UAS-Control^{Ri}] animals, and individuals that express hTau, Rab7^{T22N} and Dronc^{Ri} [GMR-Gal4, UAS-hTau, UAS-Rab7^{T22N}-GFP, UAS-Dronc^{Ri}]. **g** Quantification of hTau^{421D} levels in control [GMR-Gal4, UAS-mCD8GFP], hTau expressing [GMR-Gal4, UAS-hTau, UAS-mCD8GFP, UAS-Control^{Ri}], and hTau expression together with Rab7^{T22N} [GMR-Gal4, UAS-hTau, UAS-Rab7^{T22N}-GFP, UAS-Control^{Ri}] animals, and individuals that express hTau, Rab7^{T22N} and Dronc^{Ri} [GMR-Gal4, UAS-hTau, UAS-Rab7^{T22N}-GFP, UAS-Dronc^{Ri}]. Data are presented as mean values +/- SEM. **h** Protein analyses of head lysates from control [GMR-Gal4, UAS-mCD8GFP], hTau^{D421X} expressing [GMR-Gal4, UAS-hTau^{D421X}, UAS-mCD8GFP] and hTau^{D421X} expressing together with Rab7^{T22N} [GMR-Gal4, UAS-hTau^{D421X}, UAS-Rab7^{T22N}-GFP] animals. Unpaired two-tailed *t*-test, ***p<0.001, (**c**, anti-GFP) for Rab7^{YFP} (n = 3) vs. Rab7^{YFP} plus DeGradFP (n = 3). One-Way ANOVA, ***p<0.001 (**e**) with multiple comparisons between hTau (n = 3) and hTau plus Rab7^{WT} (n = 3) (Dunnett's test p=0.9999), hTau and hTau plus Rab7^{Q67L} (n = 3) (Dunnett's test p=0.9983), and hTau and hTau plus Rab7^{T22N} (n = 3) (Dunnett's test ***p<0.001). Unpaired two-tailed *t*-test, ***p<0.001 (**g**, hTau^{421D}) for hTau plus Rab7^{T22N} (n = 3) vs. hTau plus Rab7^{T22N} plus Dronc^{Ri} (n = 3) (n = 3 for hTau). n indicates independent biological replicates. Source data are provided as a Source Data file.

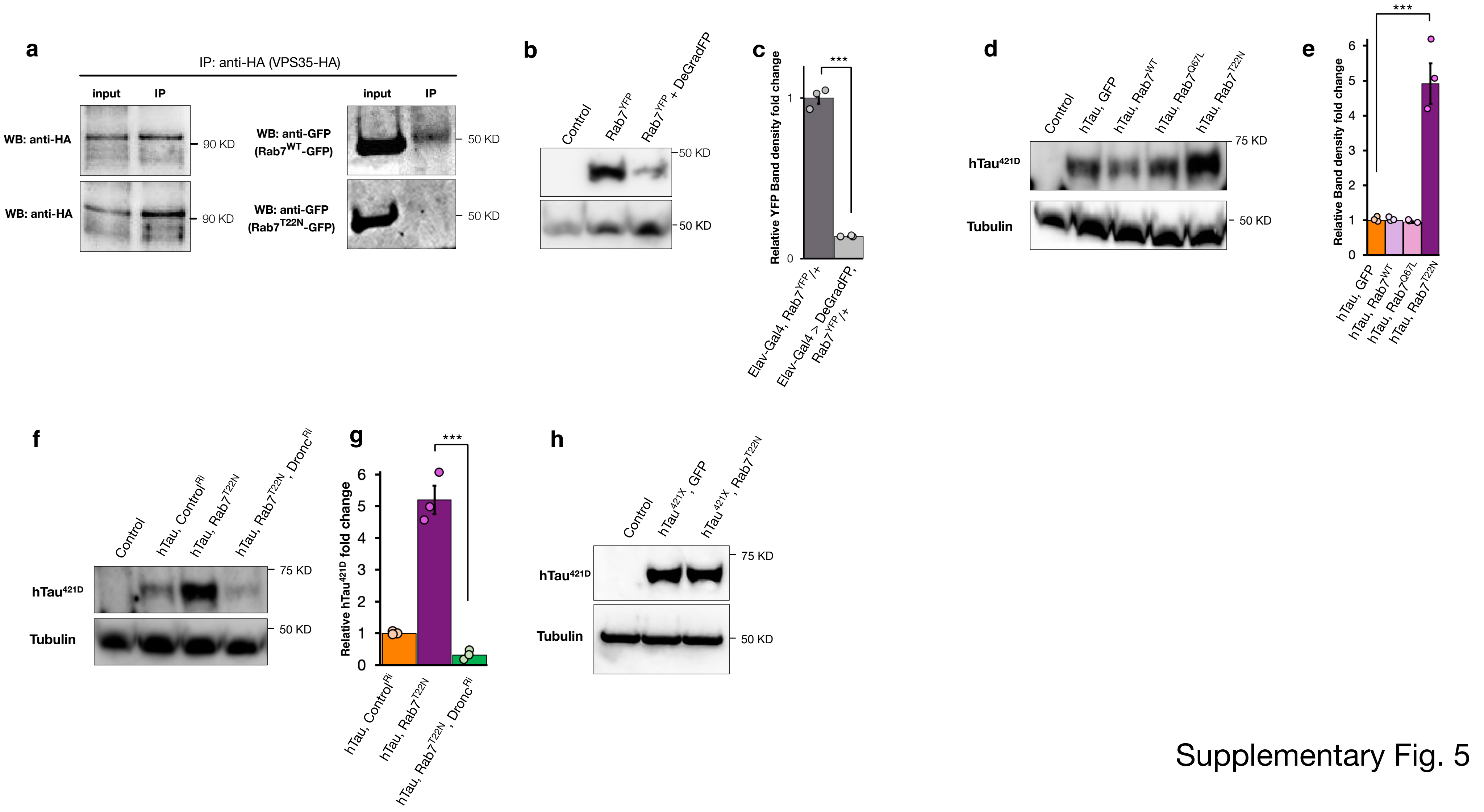

Supplementary Fig. 5

Supplementary Fig. 6 Interaction of Rab7 with Tau and hTau^{421X} in eye and lifespan assays.

a Eye morphology in hTau^{D421E} expressing [GMR-Gal4, UAS-hTau^{D421E}, UAS-mCD8GFP] and hTau^{D421E} expressing together with Rab7^{T22N} [GMR-Gal4, UAS-hTau^{D421E}, UAS-Rab7^{T22N}-GFP] animals. **b** Eye morphology in hTau^{421X} expressing [GMR-Gal4, UAS-hTau^{421X}, UAS-mCD8GFP] and hTau^{421X} expressing together with Rab7^{T22N} [GMR-Gal4, UAS-hTau^{421X}, UAS-Rab7^{T22N}-GFP] animals. **c** Eye disruption scores for hTau^{D421E} expressing [GMR-Gal4, UAS-hTau^{D421E}, UAS-mCD8GFP], hTau^{D421E} expressing together with Rab7^{T22N} [GMR-Gal4, UAS-hTau^{D421E}, UAS-Rab7^{T22N}-GFP], hTau^{421X} expressing [GMR-Gal4, UAS-hTau^{421X}, UAS-mCD8GFP], and hTau^{421X} expressing together with Rab7^{T22N} [GMR-Gal4, UAS-hTau^{421X}, UAS-Rab7^{T22N}-GFP] animals. Data are presented as mean values +/- SEM. **d** Eye morphology in hTau expressing [GMR-Gal4, UAS-hTau, UAS-mCD8GFP], hTau expressing together with Rab7^{WT} [GMR-Gal4, UAS-hTau, UAS-Rab7^{WT}], and hTau expressing together with Rab7^{Q67L} [GMR-Gal4, UAS-hTau, UAS-Rab7^{Q67L}] animals. **e** Eye disruption scores for hTau expressing [GMR-Gal4, UAS-hTau, UAS-mCD8GFP], hTau expressing together with Rab7^{WT} [GMR-Gal4, UAS-hTau, UAS-Rab7^{WT}], and hTau expressing together with Rab7^{Q67L} [GMR-Gal4, UAS-hTau, UAS-Rab7^{Q67L}] animals. Data are presented as mean values +/- SEM. **f** Survival analyses of animals expressing Rab7^{T22N} alone [*dTau*-Gal4, UAS-Rab7^{T22N}, *tubulin*-Gal80^{ts}] compared to controls [*dTau*-Gal4, UAS-mCD8GFP, *tubulin*-Gal80^{ts}]. **g** Survival analyses of hTau expressing [*dTau*-Gal4, UAS-hTau, UAS-mCD8GFP, *tubulin*-Gal80^{ts}] versus hTau together with Rab7^{T22N} expressing [*dTau*-Gal4, UAS-hTau, UAS-Rab7^{T22N}, *tubulin*-Gal80^{ts}] animals. Mann-Whitney test, p=0.4051 for eye disruption scores comparison between hTau^{D421E} (n = 33) and hTau^{D421E} plus Rab7^{T22N} (n = 33) (**c**). Mann-Whitney test, p=0.0834 for eye disruption scores comparison between hTau^{421X} (n = 31) and hTau^{421X} plus Rab7^{T22N} (n = 30) (**c**). Kruskal-Wallis test, p=0.7706 for eye disruption scores across all genotypes with multiple comparisons between hTau (n = 26) and hTau plus Rab7^{WT} (n = 30) (Dunnett's test p=0.6522), and hTau and hTau plus Rab7^{Q67L} (n = 30) (Dunnett's test p=0.9307) (**e**). Mantel-Cox test, ***p<0.001 for survival analysis of control (n = 202) vs. Rab7^{T22N} (n = 144) (**f**). Mantel-Cox test, ***p<0.001 for survival analysis of hTau (n = 192) vs. hTau plus Rab7^{T22N} (n = 193) (**g**). n indicates independent biological replicates. Source data are provided as a Source Data file.

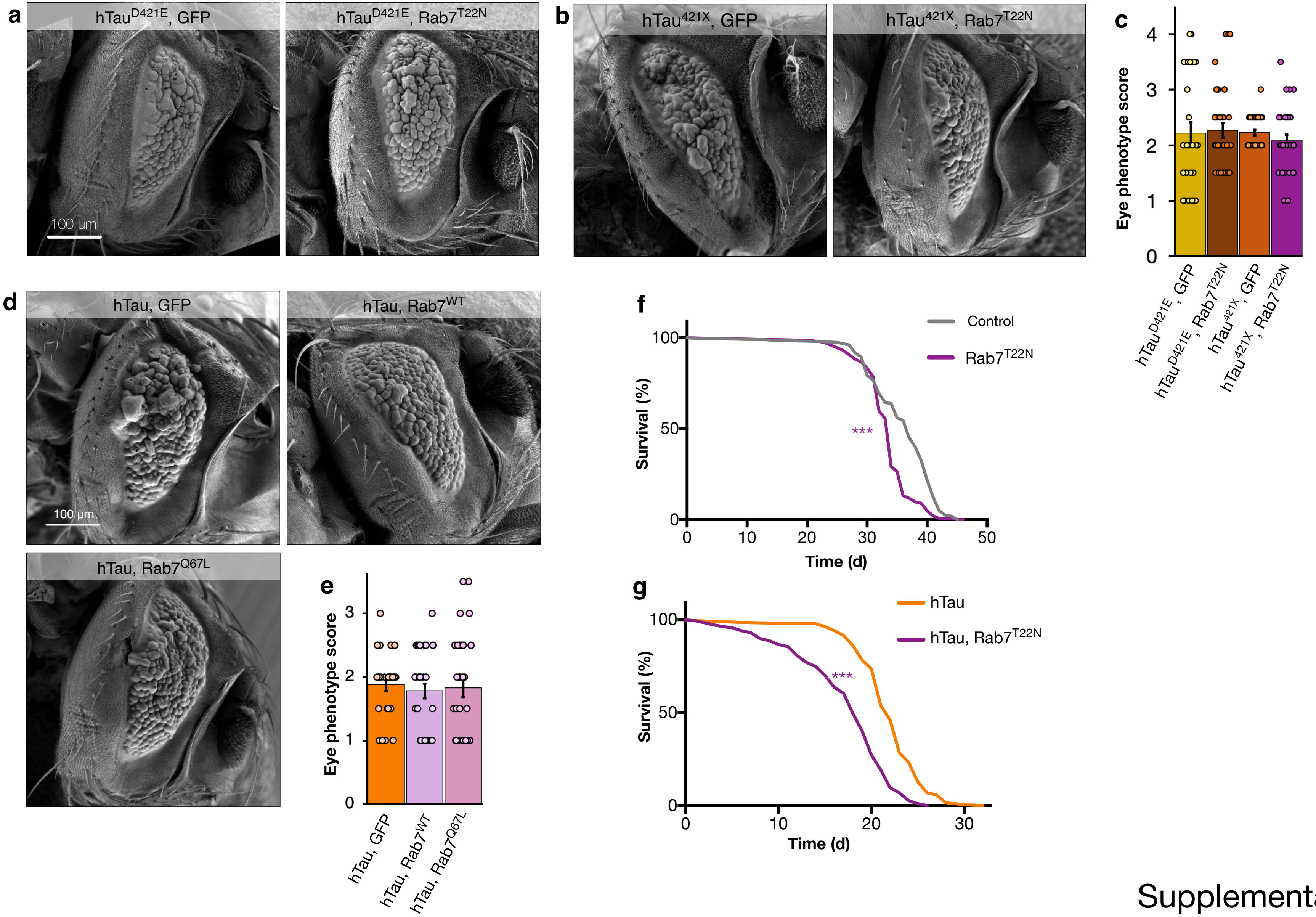

Supplementary Fig. 6

Supplementary Methods

Drosophila strains

The following lines were used in this study: GMR-Gal4[1], *ato*-Gal4 14a[2] (BDSC# 6480), *ato*-LexA[3], *dHB9*-Gal4[4], *dHB9*-QF2[4,5] (this study), *elav*-Gal4[6] (BDSC# 458), *Tubulin*-Gal80$^{[ts]}$[7], UAS-hTau[8], UAS-hTau^{421X} (this study), UAS-hTau^{D421E} (this study), dTau-Gal4 (this study), UAS-RNAi-Vps35[9] (BDSC# 38944), UAS-RNAi-Vps29[9] (BDSC# 53951), UAS-RNAi-Vps26[9] (BDSC# 38937), UAS-RNAi-Dronc[9–11] (BDSC# 32963), UAS-RNAi-Drice[9] (BDSC# 23403), UAS-RNAi-Dredd[9] (BDSC# 34070), TI{TI}Rab7$^{[EYFP]}$[12] (BDSC# 62545), UAS-DeGradFP[13] (gift from Gary Struhl), Df(3R)Exel6196 (deficiency covering Rab7, BDSC# 7675), UAS-hVPS35 (BDSC# 65810), UAS-hVPS26A[14], UAS-hVPS29 (this study), UAS-dsRNA-GFP[9], UAS-Vps35-HA[14], UAS-Vps29-HA[15] (FlyORF# F002397), UAS-Vps26-V5[14], UAS-GFP, UAS-Dronc-GFP (BDSC# 56759), UAS-His2A-GFP, QUAS-His2B-Cherry (this study), UAS-Syt1-GFP, UAS-mCherry [6X][16], UAS-Rab7-GFP (BDSC# 42705 and 42706), UAS-YFP-Rab7$^{[T22N]}$[17] (BDSC# 23235), UAS-YFP-Rab4 (BDSC# 9767)[17], UAS-YFP-Rab5[17] (BDSC# 24616), UAS-GFP-LAMP (BDSC# 42714), UAS-Venus-Rab7$^{[WT]}$[18], UAS-Venus-Rab7$^{[Q67L]}$[18], tau KO[19].

Eye phenotypes and scoring

All flies were reared at 25°C and eyes analysed within 2 days after eclosion. GMR-Gal4 was used for eye expression of transgenes. Adult eyes were imaged and recorded using a Leica MZ 16 1FA stereo microscope and scored blind to genotype, as described previously [8] with modifications to the scoring criteria as follows: (0) wild-type, (1) mild roughness and distinguishable malformed and fused ommatidia with size reduced to ≥ 60% of that of wild-type with occasional black lesions, (2) more pronounced reduction of size (≥ 50% of wild-type), more frequent black lesions and obvious ommatidia malformation and fusion, (3) size reduced to ≥ 35% of wild-type, have frequent black lesions and difficult to distinguish fused ommatidia boundries, (4) narrow 'bar-shaped' eyes (≥ 25% of wild-type size), severe ommatidia fusion with most boundaries undistinguishable and frequent large black lesions, (5) eye size severely reduced (≥ 15-20% of wild-type), no observable ommatidia

boundaries, consistent occurrence of large black lesions, (6) very small and severely malformed eyes (≥ 10% wild-type size), complete disruption of organization with no distinguishable boundaries, often consisting of single black lesion. A minimum of 20 individual animals were scored per group. All scoring data are provided in Table S2.

Scanning Electron Microscopy

Animals were anesthetized with CO2 and fixed with 2.5% glutaraldehyde for two hours at room temperature. Preparations were then washed three times with 0.1M cacodylate buffer for two minutes each and postfixed with 1% osmium tetroxide for thirty minutes. The samples were washed twice with H_2O for three minutes each, and dehydrated through an ethanol series (1x30%, 1x50%,1x 70%,1x 90%, 1x95% and 2x100%). The preparations were then dried at critical point and sputter-coated with 10 nm gold-palladium and imaged with a Zeiss Merlin Scanning Electron Microscope.

Cell loss assays

Cell death was measured by counting the number of DC neurons soma labelled by a fluorescent protein (6x-mCherry). To check the reliability of cell counting, DC neuron nuclei were also independently labelled by Histone-GFP and counted in hTau expressing animals (Figure S1C, D). Results were similar to 6x-mCherry soma count.

Adult and larval brain immunostaining

Adult brain dissections were performed according to Williamson et al.[20] In brief, adult brains were dissected in PBS solution and were then transferred to 4% formaldehyde in PBS and fixed overnight at 4°C. The next day, the brains were permeabilized in 0.3% PBST [PBS + Triton] and were incubated with primary antibodies for 3 days and were then incubated probed with secondary antibodies overnight. Labelled brains were mounted in ProLong Gold (Thermo Fisher). Quantification of DC neuron medulla axon coverage was performed using the threshold tool in ImageJ[21]. Briefly, medulla areas were cropped from Z projections of brain images. Pixel intensity was then set such that axons were above threshold. The percent medulla area occupied by axons was then measured.

Data are provided in Table S2. Larval brain dissections were performed essentially as described elsewhere[22] with the following modifications: larvae were dissected in cold PBS, fillets were prepared without removing the brain and fixed in Bouin's solution for 5 minutes. After staining with primary and secondary antibodies, the brains were dissociated from the carcass and mounted in ProLong Gold (Thermo Fisher). For staining of truncated hTau (hTau-421D), we empirically found 0.1% PBST for incubation and washes avoided disruption of intracellular organelles including endosomes. The following primary and secondary antibodies were used: chicken anti-GFP (Abcam; #ab13970) 1:1000, rabbit anti-DsRed (Clontech; #652494) 1:1000, mouse anti-Brp (DSHB; #nc82-C) 1:100, mouse anti-truncated-Tau (Asp421) clone C3 (Millipore; 36-017) 1:250, goat anti-chicken Alexa Fluor 488 (Thermo Fisher) 1:1000, donkey anti-rabbit Alexa Fluor 555 (Thermo Fisher) 1:1000, goat anti-mouse Cy5 (Jackson ImmunoResearch) 1:1000.

Medulla synapse counting

Drosophila brain images were maximum-projected in ImageJ/Fiji[23] and the outline of the medulla was user drawn. The GFP channel was extracted and smoothed with a 3x3 gaussian kernel. Synapses were detected as points using ImageJ's find Maxima plugin (https://imagej.net/ij/docs/guide/146-29.html#toc-Subsection-29.4) with a tolerance of 15. From the steps above, the number of maxima present in the GFP signal was extracted. These results were then analysed and plotted.

Colocalization analysis

Colocalization analysis was performed in ImageJ/Fiji[23]. Rab and Tau^{421D} channels were first median filtered in XY (radius = 5 pixels) and then downscaled with averaging to a voxel size of 45x45x560 nm to reduce the noise and fasten the processing. Neurons were annotated manually, and to assess colocalization between Rab and Tau^{421D} proteins, Mander's coefficients[24] were computed for each neuron, using "Moments" automated threshold, based on the z-stack histogram of each channel (https://imagej.net/Auto_Threshold). The plot shown in Fig. 6d displays the pair of Mander's

coefficient for each neuron (M1 reflects the fraction of Rab signal overlapping Tau^{421D} signal, while M2 reflects the fraction of Tau^{421D} signal overlapping with Rab signal).

Immunoprecipitation

We employed GMR-Gal4 to co-express UAS-Vps35-HA together with UAS-Venus-Rab7^{WT} or UAS-Venus-Rab7^{T22N}. Heads were homogenized in ice-cold lysis buffer (25 mM Tris–HCl [pH 7.5], 150 mM NaCl, 5 mM EDTA, 1% [v/v] NP-40, 5% [v/v] glycerol) containing fresh protease and phosphatase inhibitors (Roche). After homogenization, samples were centrifuged at 1,200 x g for 5 min at 4°C and supernatants were centrifuged again at 13,000 x g for 5 min at 4°C. Supernatants were then pre-cleared with A/G sepharose beads (Sigma-Aldrich) for 30 min at 4°C and incubated with primary antibody overnight at 4°C. Next day, samples were incubated with A/G sepharose beads for two hours before addition of sample buffer and elusion at 95°C for 5 min. The immunoprecipitated material were then loaded on western blot gels for analysis. We used mouse anti-HA (Covance) for Co-IP (1:100) and for western blot (1:1000) and rabbit anti-GFP (Cell Signalling) (1:4000) for western blots.

Protein measurements

Protein measurements were performed using adult head lysates. Heads were collected from the desired genotype and homogenized in RIPA buffer (140mM NaCl, 1mM EDTA, 0.5 mM EGTA, 1% Triton X-100, 10mM Tris-Cl PH8.0) containing freshly-added protease and phosphatase inhibitors (Roche). Lysates were then reduced in sample buffer and an equal amount of total protein was loaded and electrophoresed on 4-12% gradient precast gels (Thermo Fisher, XP04120BOX) and then transferred to PVDF membrane using an iBlot 2 Dry Blotting System (Life Technologies). Blots were then fixed in Ponceau S solution (Sigma-Aldrich), blocked in milk for 1 hour and incubated with primary antibodies overnight. The next day blots were washed in 0.1% PBST, incubated with secondary antibodies for one hour and treated with WesternBright Sirius detection kit (advansta, K-12043-C20) for 5 minutes before imaging using a Fusion FX (Vilber Lourmat) or an Amersham

Imager 680. We empirically found that the following modifications enhanced the quality of blots probed with anti-hTau^{421D} (a) a brief low-power sonication (<10 seconds) of samples immediately after homogenization and before addition of sample buffer, (b) overnight treatment of blots in ponceau S solution at 4°C, and (c) analysis of proteins of only > 45 KD. Densitometric analyses of western blots were performed using Gels tool in ImageJ (https://imagej.nih.gov/ij/). The following antibodies were used: mouse anti-cleaved-Tau (Asp421) clone C3 (Millipore; 36-017) 1:1000, rabbit anti-TauC (1:20,000), rabbit anti-PHF-1 (Sigma) 1:2000, monoclonal anti-phospho-Tau (Ser202, Thr205) (AT8) (Thermo Fisher) 1:2000, monoclonal anti- phospho-Tau (Thr212, Ser214) (AT100) (Thermo Fisher) 1:2000, monoclonal anti-Spectrin (DSHB) 1:1000, monoclonal anti-alpha tubulin (Sigma) 1:2000, mouse anti-HA (Covance) 1:1000, guinea pig anti-Vps26 (gift from Hugo Bellen) (1:4000), HRP anti-mouse (Jackson Immunoresearch) (1:20,000), HRP anti-rabbit (Jackson ImmunoResearch) (1:20,000), HRP anti-guinea pig (1:20,000).

Longevity assay

Crosses were established and kept at 18°C. Adult animals were collected 0-8 hours post eclosion and were maintained in vials of ≤10 at 18°C for an additional 2 days. These animals were then transferred to 29°C when measurements of lifespan were initiated. Flies were transferred to fresh vials every second day and throughout the experiment the vials were laid on their side to minimize death due to falling on the food. A total of 150 – 200 flies were assayed for each genotype. Statistical analyses for lifespan were carried out using Prism 7.0a and the inbuilt Log-rank (Mantel-Cox) test.

Cell culture

Immortalized CN1.4 mouse cortical neurons (CN) that stably express inducible forms of full-length human tau (2N4R) were described previously[25]. Cells were plated at an average density of 3.6 × 103 cells/cm2 and maintained in DMEM/F12 medium supplemented with 10% FCS US origin (Sigma-Aldrich), 50mg/ml Hygromycin (Invitrogen) at 32°C, 5% CO2. 24h after plating, lentiviruses were added at a concentration of 430 transfusing unit (TU) /cells together with 2ug/ml DEAE-Dextran HCl. 48h later, Tau expression was induced as follow: First, pre-induction was done by direct application

of 1mg/ml Doxycycline and incubated for 14h at 32°C, 5% CO2. Next, cell medium was exchanged against DMEM/F12, 1% FBS, 50 ug/mL Hygromycin and 1ug/ml Doxycycline, cells were transferred at 37°C, 5% CO2 and incubated for 3 days.

Lentivirus preparation

Scrambled shRNA lentivirus pGFP-C-shLenti (TL500450, Origene) was a generous gift from Professor Johannes Graff. All other lentiviruses were purified by Bertarelli Foundation Gene Therapy Platform (Switzerland). The following sequences and clones were used for generation of lentiviruses used in this study: CCATCAGAGGATGTTGTATC for shRNA-Vps35, TRC Lentiviral Mouse Vps26a shRNA, clone id: TRCN0000115332 (RMM3981-201833326, Horizon Discovery Ltd), TRC Lentiviral Mouse Vps29 shRNA, clone id: TRCN0000111589 (RMM3981-201826859, Horizon Discovery Ltd).

CN 1.4 cells transduction

Briefly, immortalized CN1.4 mouse cortical neurons were plated at $0.25x10^6$ cell/cm^2 and infected the next day with lentiviral particles for expressing Vps35 shRNA or scrambled shRNA. After 48 hours, cells were treated with 1µg/ml of doxycycline (Roche) for 48h to induce hTau transgene expression.

Cell lysis, and immunoblotting

Cells were harvested in lysis buffer (137 mM NaCl, 20 mM Tris·HCl [pH 7.5], 1% Nonidet P-40, and 10% [vol/vol] glycerol, Complete Ultra, protease inhibitor [Roche] and PhosStop [Roche]). Cell extracts were sonicated three times for 5 s on ice and cell debris were removed by centrifugation at 12,000 × g for 10 min at 4 °C. Loading buffer (giving rise to a final concentration of 10% glycerol, 2% [wt/vol] SDS, 5% [vol/vol] β-Mercaptoethanol, and 0.01% bromophenol blue) was added to 30 µg protein samples before denaturation step at 70 °C for 10 min. Proteins were resolved on WedgeWells 4-12% Bis-Tris minigels using MES–SDS running buffer (Invitrogen) and transferred to iBlot PVDF membranes (Invitrogen). Membranes were blocked for 1 h in PBST (PBS, 0.1% Triton X-100) and

5% (wt/vol) nonfat dried milk (Applichem). Antibody directed against truncated Tau X-421 (Biolegend) was diluted 1/200 in PBST-5% milk. Incubation with primary antibody was done overnight at 4 °C. After washing with PBST, the blots were probed with the appropriate horseradish peroxidase conjugated secondary antibody diluted 1/20,000 in 2.5% PBST-2.5% milk. After extensive washing with PBST, the peroxidase activity was detected by chemiluminescence using WesternBright Sirius (Advansta) and the Amersham Imager 680 (GE Healthcare). To ensure equal loading, PVDF membranes were stripped (Restore stripping reagent; Pierce) for 10 min at room temperature, washed several times with PBST, and re-probed as described above using a mouse monoclonal antibody directed against Tubulin (Sigma-Aldrich), diluted at 1/20,000. Protein expression was quantified using ImageJ software and bands were normalized to their corresponding Tubulin content. Results are expressed as percentage of control values.

Quantitative RT PCR

Total RNA was isolated from cultured cells using Nucleospin RNA II kit (Macherey-Nagel) according to the manufacturer's instructions. The first strand of cDNA was synthesized from 100 ng of total RNA (60 min at 37 °C followed by 5 min at 95 °C) using High-Capacity RNA-to-cDNA kit (Applied Biosystems). The resulting cDNA was amplified by quantitative PCR with an QuantStudio 6 Flex Real-Time PCR system (Thermo Fisher Scientific Inc). The PCR mix was composed of 6 ng of cDNA, 300 nM of forward and reverse primers in 10 µL of 1× Power SYBR-Green PCR MasterMix (Applied Biosystems). Primer sequences were designed using PrimerQuest online tool (Integrated DNA technologies, Inc.) and oligonucleotides were synthesized by Microsynth AG (Switzerland). For adult dTau mRNA quantifications, total RNA extract was isolated from 3 adult flies (N: 9 RNA extract over 3 independent experiments for each condition). Forward and reverse sequences were as follows: β-Actin (NM_007393): 5′- GCTTCTTTGCAGCTCCTTCGT-3′ and 5′-ATATCGTCATCCATGGCGAAC-3′; Cyclophilin A (NM_008907): 5′-CAAATGCTGGACCAAACACAA-3′ and 5′-GCCATCCAGCCATTCAGTCT-3′; VPS26a (NM_ 133672): 5′- CCACGATGAGAGATGTGAA-3′ and 5′- GATCTCCTGCTGCTTGAA -3′; VPS29

(NM_019780): 5′- GGATGTTGGTGTTGGTACT-3′ and 5′- CAGAGGATGTGCTGGATTT-3′; VPS35 (NM_022997): 5′- CTCTCAGGACCAGGTAGATT -3′ and 5′- TCTGGATCAGGGTCTTCTAC -3′; dTau (NM_170345) 5′- GAGCCAGTGAAGAAAGTTCCA -3′ and 5′- CGGCTTATAGGTGGCATTGT -3′. The specificity of PCR amplification for each set of primers was checked by the presence of a single sharp peak in the melting curve analysis. Data were computed using the sequence detector software QuantStudio v1.7.1 (Thermo Fisher Scientific Inc.) and analyzed using a macro developed by the genomic platform of Geneva University (Frontiers in Genetics, University of Geneva). Delta-Ct relative quantification (ΔΔCt) with cyclophilin and actin as reference genes was used. For each mRNA transcript, data were expressed as fold changes relative to the control condition.

Supplementary Methods References

1. Ellis, M. C., O'Neill, E. M. & Rubin, G. M. Expression of Drosophila glass protein and evidence for negative regulation of its activity in non-neuronal cells by another DNA-binding protein. *Development* **119**, 855–865 (1993).
2. Hassan, B. A. *et al.* Atonal regulates neurite arborization but does not act as a proneural gene in the Drosophila brain. *Neuron* **25**, 549–561 (2000).
3. Langen, M. *et al.* Mutual inhibition among postmitotic neurons regulates robustness of brain wiring in Drosophila. *Elife* 1–21 (2013). doi:10.7554/eLife.00337
4. Broihier, H. T. & Skeath, J. B. Drosophila homeodomain protein dHb9 directs neuronal fate via crossrepressive and cell-nonautonomous mechanisms. *Neuron* **35**, 39–50 (2002).
5. Lin, C. C. & Potter, C. J. Editing transgenic DNA components by inducible gene replacement in Drosophila melanogaster. *Genetics* **203**, 1613–1628 (2016).
6. Robinow, S. & White, K. The locus elav of Drosophila melanogaster is expressed in neurons at all developmental stages. *Dev. Biol.* **126**, 294–303 (1988).
7. McGuire, S. E., Mao, Z. & Davis, R. L. Spatiotemporal Gene Expression Targeting with the

TARGET and Gene-Switch Systems in Drosophila. *Sci. Signal.* **2004**, pl6–pl6 (2004).

8. Santa-Maria, I. *et al.* Dysregulation of microRNA-219 promotes neurodegeneration through post-transcriptional regulation of tau. *J. Clin. Invest.* **125**, 681–686 (2015).
9. Ni, J. Q. *et al.* A genome-scale shRNA resource for transgenic RNAi in Drosophila. *Nat. Methods* **8**, 405–407 (2011).
10. Amcheslavsky, A. *et al.* Plasma Membrane Localization of Apoptotic Caspases for Non-apoptotic Functions. *Dev. Cell* **45**, 450-464.e3 (2018).
11. Chi, C., Wang, L., Lan, W., Zhao, L. & Su, Y. PpV, acting via the JNK pathway, represses apoptosis during normal development of Drosophila wing. *Apoptosis 2018 239* **23**, 554–562 (2018).
12. Dunst, S. *et al.* Endogenously Tagged Rab Proteins: A Resource to Study Membrane Trafficking in Drosophila. *Dev. Cell* **33**, 351–365 (2015).
13. Harmansa, S., Alborelli, I., Caussinus, E. & Affolter, M. A nanobody-based toolset to investigate the role of protein localization and dispersal in Drosophila. *bioRxiv* 1–22 (2017). doi:10.7554/eLife.22549
14. Wang, S. *et al.* The Retromer Complex Is Required for Rhodopsin Recycling and Its Loss Leads to Photoreceptor Degeneration. *PLoS Biol.* **12**, (2014).
15. Bischof, J., Sheils, E. M., Björklund, M. & Basler, K. Generation of a transgenic ORFeome library in Drosophila. *Nat. Protoc.* **9**, 1607–1620 (2014).
16. Shearin, H. K., MacDonald, I. S., Spector, L. P. & Steven Stowers, R. Hexameric GFP and mCherry reporters for the Drosophila GAL4, Q, and LexA transcription systems. *Genetics* **196**, 951–960 (2014).
17. Zhang, J. *et al.* Thirty-one flavors of Drosophila Rab proteins. *Genetics* **176**, 1307–1322 (2007).
18. Cherry, S. *et al.* Charcot-Marie-Tooth 2B mutations in rab7 cause dosage-dependent neurodegeneration due to partial loss of function. *Elife* **2013**, 1–22 (2013).
19. Burnouf, S. *et al.* Deletion of endogenous Tau proteins is not detrimental in Drosophila. *Sci. Rep.* **6**, 23102 (2016).

20. Williamson, W. R. & Hiesinger, P. R. Preparation of Developing and Adult Drosophila Brains and Retinae for Live Imaging. *J. Vis. Exp.* (2010). doi:10.3791/1936

21. Jaerve, A., Schiwy, N., Schmitz, C. & Mueller, H. W. Differential effect of aging on axon sprouting and regenerative growth in spinal cord injury. *Exp. Neurol.* **231**, 284–294 (2011).

22. Brent, J., Werner, K. & McCabe, B. D. Drosophila Larval NMJ Immunohistochemistry. *J. Vis. Exp.* (2009).

23. Schindelin, J. *et al.* Fiji: an open-source platform for biological-image analysis. *Nat. Methods* **9**, 676–682 (2012).

24. Manders, E. M. M., Verbeek, F. J. & Aten, J. A. Measurement of co-localization of objects in dual-colour confocal images. *J. Microsc.* **169**, 375–382 (1993).

25. Matthews-Roberson, T. A., Quintanilla, R. A., Ding, H. & Johnson, G. V. W. Immortalized cortical neurons expressing caspase-cleaved tau are sensitized to endoplasmic reticulum stress induced cell death. *Brain Res.* **1234**, 206–212 (2008).

Supplementary Table 1
Expanded figure genotypes

Figure	Figure Label	Full Genotype
1a	Control	GMR-Gal4, UAS-mCD8GFP
1a	hTau	GMR-Gal4, UAS-hTau, UAS-Control^{Ri}
1c	dTau-Gal4, UAS-mCherry-CAAX	dTau-Gal4, UAS-mCherry-CAAX
1d	Control	dTau-Gal4, UAS-mCD8GFP, tubulin-Gal80^{ts}
1d	hTau	dTau-Gal4, UAS-hTau, tubulin-Gal80^{ts}
1f		Ato-Gal4, UAS-mCD8GFP
1g	Control	Ato-Gal4, UAS-mCherry, UAS-mCD8GFP, UAS-Syn-GFP, tubulin-Gal80^{ts}
1g	hTau	Ato-Gal4, UAS-hTau, UAS-mCherry, UAS-Syn-GFP, tubulin-Gal80^{ts}
1i	Control	Ato-Gal4, UAS-mCherry, UAS-mCD8GFP, tubulin-Gal80^{ts}
1i	hTau	Ato-Gal4, UAS-hTau, UAS-mCherry, tubulin-Gal80^{ts}
2a	Control	GMR-Gal4, UAS-mCD8GFP
2a	hTau,Control^{Ri}	GMR-Gal4, UAS-hTau, UAS-Control^{Ri}
2a	hTau, Vps35^{Ri}	GMR-Gal4, UAS-hTau, UAS-Vps35^{Ri}
2a	hTau, Vps29^{Ri}	GMR-Gal4, UAS-hTau, UAS-Vps29^{Ri}
2a	hTau, Vps26^{Ri}	GMR-Gal4, UAS-hTau, UAS-Vps26^{Ri}
2c	hTau	dTau-Gal4, UAS-hTau, UAS-Control^{Ri}, tubulin-Gal80^{ts}
2c	hTau, Vps35^{Ri}	dTau-Gal4, UAS-hTau, UAS-Vps35^{Ri}, tubulin-Gal80^{ts}
2c	hTau, Vps29^{Ri}	dTau-Gal4, UAS-hTau, UAS-Vps29^{Ri}, tubulin-Gal80^{ts}
2c	hTau, Vps26^{Ri}	dTau-Gal4, UAS-hTau, UAS-Vps26^{Ri}, tubulin-Gal80^{ts}
2d	hTau	Ato-Gal4, UAS-hTau, UAS-mCD8GFP, UAS-mCherry, tubulin-Gal80^{ts}
2d	hTau, Vps35^{Ri}	Ato-Gal4, UAS-hTau, UAS-Vps35^{Ri}, UAS-mCherry, tubulin-Gal80^{ts}
2d	hTau, Vps29^{Ri}	Ato-Gal4, UAS-hTau, UAS-Vps29^{Ri}, UAS-mCherry, tubulin-Gal80^{ts}
2d	hTau, Vps26^{Ri}	Ato-Gal4, UAS-hTau, UAS-Vps26^{Ri}, UAS-mCherry, tubulin-Gal80^{ts}
3a	Control	GMR-Gal4, UAS-mCD8GFP
3a	hTau,Control^{Ri}	GMR-Gal4, UAS-hTau, UAS-Control^{Ri}
3a	hTau, Vps26^{Ri}	GMR-Gal4, UAS-hTau, UAS-Vps26^{Ri}

Supplementary Table 1
Expanded figure genotypes

3a	hTau, $Vps29^{Ri}$	GMR-Gal4, UAS-hTau, UAS-$Vps29^{Ri}$
3a	hTau, $Vps35^{Ri}$	GMR-Gal4, UAS-hTau, UAS-$Vps35^{Ri}$
3e	Control	GMR-Gal4, UAS-mCD8GFP
3e	hTau,$Control^{Ri}$	GMR-Gal4, UAS-hTau, UAS-$Control^{Ri}$
3e	hTau, $Dronc^{Ri}$	GMR-Gal4, UAS-hTau, UAS-$Dronc^{Ri}$
3g	Control	Ato-Gal4, UAS-mCherry, UAS-mCD8GFP, tubulin-$Gal80^{ts}$
3g	hTau	Ato-Gal4, UAS-hTau, UAS-mCherry, UAS-mCD8GFP, tubulin-$Gal80^{ts}$
3g	hTau, $Dronc^{Ri}$	Ato-Gal4, UAS-hTau, UAS-$Dronc^{Ri}$, UAS-mCherry, tubulin-$Gal80^{ts}$
4a	Control	GMR-Gal4, UAS-mCD8GFP
4a	hTau	GMR-Gal4, UAS-hTau
4a	$hTau^{D421E}$	GMR-Gal4, UAS-$hTau^{D421E}$
4b	Control	GMR-Gal4, UAS-mCD8GFP
4b	$hTau^{D421E}$, $Control^{Ri}$	GMR-Gal4, UAS-$hTau^{D421E}$, $Control^{Ri}$
4b	$hTau^{D421E}$, $Vps35^{Ri}$	GMR-Gal4, UAS-$hTau^{D421E}$, UAS-$Vps35^{Ri}$
4b	$hTau^{D421E}$, $Vps29^{Ri}$	GMR-Gal4, UAS-$hTau^{D421E}$, UAS-$Vps29^{Ri}$
4b	$hTau^{D421E}$, $Vps26^{Ri}$	GMR-Gal4, UAS-$hTau^{D421E}$, UAS-$Vps26^{Ri}$
4d	$hTau^{D421E}$, $Control^{Ri}$	dTau-Gal4, UAS-$hTau^{D421E}$, UAS-$Cherry^{Ri}$, tubulin-$Gal80^{ts}$
4d	$hTau^{D421E}$, $Vps35^{Ri}$	dTau-Gal4, UAS-$hTau^{D421E}$, UAS-$Vps35^{Ri}$, tubulin-$Gal80^{ts}$
4d	$hTau^{D421E}$, $Vps29^{Ri}$	dTau-Gal4, UAS-$hTau^{D421E}$, UAS-$Vps29^{Ri}$, tubulin-$Gal80^{ts}$
4d	$hTau^{D421E}$, $Vps26^{Ri}$	dTau-Gal4, UAS-$hTau^{D421E}$, UAS-$Vps26^{Ri}$, tubulin-$Gal80^{ts}$
4e	$hTau^{D421E}$	Ato-Gal4, UAS-$hTau^{D421E}$, UAS-mCherry, UAS-mCD8GFP, tubulin-$Gal80^{ts}$
4e	$hTau^{D421E}$, $Vps35^{Ri}$	Ato-Gal4, UAS-$hTau^{D421E}$, UAS-$Vps35^{Ri}$, UAS-mCherry, tubulin-$Gal80^{ts}$
4e	$hTau^{D421E}$, $Vps29^{Ri}$	Ato-Gal4, UAS-$hTau^{D421E}$, UAS-$Vps29^{Ri}$, UAS-mCherry, tubulin-$Gal80^{ts}$
4e	$hTau^{D421E}$, $Vps26^{Ri}$	Ato-Gal4, UAS-$hTau^{D421E}$, UAS-$Vps26^{Ri}$, UAS-mCherry, tubulin-$Gal80^{ts}$
5a	Control	GMR-Gal4, UAS-mCD8GFP
5a	$hTau^{421X}$, $Control^{Ri}$	GMR-Gal4, UAS-$hTau^{421X}$, UAS-$Control^{Ri}$
5a	$hTau^{421X}$, $Vps35^{Ri}$	GMR-Gal4, UAS-$hTau^{421X}$, UAS-$Vps35^{Ri}$

Supplementary Table 1
Expanded figure genotypes

5a	hTau^{421X}, Vps29^{Ri}	GMR-Gal4, UAS-hTau^{421X}, UAS-Vps29^{Ri}
5a	hTau^{421X}, Vps26^{Ri}	GMR-Gal4, UAS-hTau^{421X}, UAS-Vps26^{Ri}
5c	Control	GMR-Gal4, UAS-mCD8GFP
5c	hTau^{421X}, Control^{Ri}	GMR-Gal4, UAS-hTau^{421X}, UAS-Control^{Ri}
5c	hTau^{421X}, Vps35^{Ri}	GMR-Gal4, UAS-hTau^{421X}, UAS-Vps35^{Ri}
5c	hTau^{421X}, Vps29^{Ri}	GMR-Gal4, UAS-hTau^{421X}, UAS-Vps29^{Ri}
5c	hTau^{421X}, Vps26^{Ri}	GMR-Gal4, UAS-hTau^{421X}, UAS-Vps26^{Ri}
6a	hTau, mCherry	HB9-Gal4, UAS-hTau, UAS-mCherry, UAS-mCD8GFP
6b	hTau, mCherry	HB9-Gal4, UAS-hTau, UAS-mCherry, UAS-mCD8GFP
6c	hTau, Rab7-GFP, mCherry	HB9-Gal4, UAS-hTau, UAS-mCherry, UAS-Rab7-GFP
6e	hTau, Vps26^{Ri}, mCherry	HB9-Gal4, UAS-hTau, UAS-mCherry, UAS-Vps26^{Ri}
6f	hTau, Vps26^{Ri}, mCherry	HB9-Gal4, UAS-hTau, UAS-mCherry, UAS-Vps26^{Ri}
6i	hTau, Vps26^{Ri}, Rab7-GFP, mCherry	HB9-Gal4, UAS-hTau, UAS-mCherry, UAS-Rab7-GFP, UAS-Vps26^{Ri}
7a	Control	GMR-Gal4, UAS-mCD8GFP
7a	hTau	GMR-Gal4, UAS-hTau, UAS-DeGradFP
7a	hTau, Rab7^{KD}	GMR-Gal4, UAS-hTau, UAS-DeGradFP, Rab7-YFP/Δrab7
7a	Control	GMR-Gal4, UAS-mCD8GFP
7a	hTau, GFP	GMR-Gal4, UAS-hTau, UAS-mCD8GFP
7a	hTau, Rab7^{T22N}	GMR-Gal4, UAS-hTau, UAS-Rab7^{T22N}
7c	Control	GMR-Gal4, UAS-mCD8GFP
7c	hTau, GFP	GMR-Gal4, UAS-hTau, UAS-mCD8GFP
7c	Rab7^{KD}	GMR-Gal4, UAS-DeGradFP, Rab7-YFP/Δrab7
7c	hTau, Rab7^{KD}	GMR-Gal4, UAS-hTau, UAS-DeGradFP, Rab7-YFP/Δrab7
7c	Rab7^{T22N}	GMR-Gal4, UAS-Rab7^{T22N}, UAS-mCD8GFP
7c	hTau, Rab7^{T22N}	GMR-Gal4, UAS-hTau, UAS-Rab7^{T22N}

Supplementary Table 1
Expanded figure genotypes

7e	Control	Ato-Gal4,UAS-mCherry, UAS-mCD8GFP, tubulin-Gal80^{ts}
7e	Rab7^{T22N}	Ato-Gal4, UAS-Rab7^{T22N}, UAS-mCherry, tubulin-Gal80^{ts}
7e	hTau	Ato-Gal4, UAS-hTau, UAS-mCherry, UAS-mCD8GFP, tubulin-Gal80^{ts}
7e	hTau, Rab7^{T22N}	Ato-Gal4, UAS-hTau, UAS-Rab7^{T22N}, UAS-mCherry, tubulin-Gal80^{ts}
S1b		Ato-Gal4, UAS-hTau, UAS-mCherry, UAS-mCD8GFP, tubulin-Gal80^{ts}
S1c		Ato-Gal4, UAS-His-GFP, UAS-mCherry, tubulin-Gal80^{ts}
S1d	Control	Ato-Gal4, UAS-His-GFP, UAS-mCherry, tubulin-Gal80^{ts}
S1d	hTau	Ato-Gal4, UAS-hTau, UAS-His-GFP, UAS-mCherry, tubulin-Gal80^{ts}
S1f	WT1118	WT1118
S1f	dTau-Gal4 / +	dTau-Gal4
S1f	dTau KO	dTau KO
S1g	dTau-Gal4-mCD8GFP, Ato-LexA-mCherry	dTau-Gal4, UAS-mCD8GFP, Ato-LexA, LexAop-mCherry, tubulin-Gal80^{ts}
S1g	dTau-Gal4-hTau, Ato-LexA-mCherry	dTau-Gal4, UAS-hTau, Ato-LexA, LexAop-mCherry, tubulin-Gal80^{ts}
S2a	VPS35-HA (Control)	Elav-Gal4, UAS-mCD8GFP, UAS-VPS35-HA
S2a	VPS35-HA (RNAi)	Elav-Gal4, UAS-VPS35-HA, UAS-Vps35^{Ri}
S2a	VPS29-HA (Control)	Elav-Gal4, UAS-mCD8GFP, UAS-VPS29-HA
S2a	VPS29-HA (RNAi)	Elav-Gal4, UAS-VPS29-HA, UAS-Vps29^{Ri}
S2a	VPS26-HA (Control)	Elav-Gal4, UAS-mCD8GFP
S2a	VPS26-HA (RNAi)	GMR-Gal4, UAS-Vps26^{Ri}
S2c	Vps35^{Ri}	GMR-Gal4, UAS-Vps35^{Ri}
S2c	Vps29^{Ri}	GMR-Gal4, UAS-Vps29^{Ri}
S2c	Vps26^{Ri}	GMR-Gal4, UAS-Vps26^{Ri}
S2d	Control	dTau-Gal4, UAS-Cherry^{Ri}, tubulin-Gal80^{ts}
S2d	Vps35^{Ri}	dTau-Gal4, UAS-Vps35^{Ri}, tubulin-Gal80^{ts}
S2d	Vps29^{Ri}	dTau-Gal4, UAS-Vps26^{Ri}, tubulin-Gal80^{ts}

Supplementary Table 1
Expanded figure genotypes

S2d	Vps26^{Ri}	dTau-Gal4, UAS-Vps29^{Ri}, tubulin-Gal80^{ts}
S2e	Control	Ato-Gal4, UAS-mCherry, UAS-mCD8GFP, tubulin-Gal80^{ts}
S2e	Vps35^{Ri}	Ato-Gal4, UAS-Vps35^{Ri}, UAS-mCherry, UAS-mCD8GFP, tubulin-Gal80^{ts}
S2e	Vps29^{Ri}	Ato-Gal4, UAS-Vps29^{Ri}, UAS-mCherry, UAS-mCD8GFP, tubulin-Gal80^{ts}
S2e	Vps26^{Ri}	Ato-Gal4, UAS-Vps26^{Ri}, UAS-mCherry, UAS-mCD8GFP, tubulin-Gal80^{ts}
S2f	Control	Ato-Gal4, UAS-mCherry, UAS-mCD8GFP, tubulin-Gal80^{ts}
S2f	VPS26-V5	Ato-Gal4, UAS- mCherry, UAS-Vps26-V5, tubulin-Gal80^{ts}
S2f	VPS29-HA	Ato-Gal4, UAS- mCherry, UAS-Vps29-HA, tubulin-Gal80^{ts}
S2f	VPS35-HA	Ato-Gal4, UAS- mCherry, UAS-Vps35-HA, tubulin-Gal80^{ts}
S3a	Control	GMR-Gal4, UAS-mCD8GFP
S3a	hTau,Control^{Ri}	GMR-Gal4, UAS-hTau, UAS-mCD8GFP, UAS-Control^{Ri}
S3a	hTau, Vps35^{Ri}	GMR-Gal4, UAS-hTau, UAS-mCD8GFP, UAS-Vps35^{Ri}
S3a	hTau, Vps35^{Ri}, hVPS35	GMR-Gal4, UAS-hTau, UAS-Vps35^{Ri}, UAS-hVPS35
S3a	Control	GMR-Gal4, UAS-mCD8GFP
S3a	hTau,Control^{Ri}	GMR-Gal4, UAS-hTau, UAS-mCD8GFP, UAS-Control^{Ri}
S3a	hTau, Vps29^{Ri}	GMR-Gal4, UAS-hTau, UAS-mCD8GFP, UAS-Vps29^{Ri}
S3a	hTau, Vps29^{Ri}, hVPS29	GMR-Gal4, UAS-hTau, UAS-Vps29^{Ri}, UAS-hVPS29
S3a	Control	GMR-Gal4, UAS-mCD8GFP
S3a	hTau,Control^{Ri}	GMR-Gal4, UAS-hTau, UAS-mCD8GFP, UAS-Control^{Ri}
S3a	hTau, Vps26^{Ri}	GMR-Gal4, UAS-hTau, UAS-mCD8GFP, UAS-Vps26^{Ri}
S3a	hTau, Vps26^{Ri}, hVPS26A	GMR-Gal4, UAS-hTau, UAS-Vps26^{Ri}, UAS-hVPS26A
S3e	Control	GMR-Gal4, UAS-mCD8GFP
S3e	hTau,Control^{Ri}	GMR-Gal4, UAS-hTau, UAS-Control^{Ri}
S3e	hTau, Drice^{Ri}	GMR-Gal4, UAS-hTau, UAS-Drice^{Ri}
S3e	hTau, Dredd^{Ri}	GMR-Gal4, UAS-hTau, UAS-Dredd^{Ri}
S3e	hTau, Dronc^{Ri}	GMR-Gal4, UAS-hTau, UAS-Dronc^{Ri}

Supplementary Table 1
Expanded figure genotypes

S3g	Control	GMR-Gal4, UAS-mCD8GFP
S3g	hTau,Control^{Ri}	GMR-Gal4, UAS-mCD8GFP, UAS-hTau, UAS-Control^{Ri}
S3g	hTau, Vps26^{Ri}	GMR-Gal4, UAS-mCD8GFP, UAS-hTau, UAS-Vps26^{Ri}
S3g	hTau, Vps26^{Ri}, Dronc^{Ri}	GMR-Gal4, UAS-hTau, UAS-Vps26^{Ri}, UAS-Dronc^{Ri}
S3i	Control	GMR-Gal4, UAS-mCD8GFP
S3i	hTau,GFP	GMR-Gal4, UAS-hTau, UAS-mCD8GFP
S3i	hTau,Dronc-GFP	GMR-Gal4, UAS-hTau, UAS-Dronc-GFP
S3k	Control	dTau-Gal4, UAS-mCD8GFP, UAS-Cherry^{Ri}, tubulin-Gal80^{ts}
S3k	Dronc^{Ri}	dTau-Gal4, UAS-mCD8GFP, UAS-Dronc^{Ri}, tubulin-Gal80^{ts}
S3l	hTau	dTau-Gal4, UAS-hTau, UAS-Cherry^{Ri}, tubulin-Gal80^{ts}
S3l	hTau, Dronc^{Ri}	dTau-Gal4, UAS-hTau, UAS-Dronc^{Ri}, tubulin-Gal80^{ts}
S4a		dTau-Gal4, UAS-His2A-GFP, dHB9-QF2, QUAS-His2B-Cherry
S4b	hTau, mCherry	dHB9-Gal4, UAS-hTau, UAS-mCherry
S4c	Rab4-GFP, hTau, mCherry	dHB9-Gal4, hTau, UAS-Rab4-GFP, UAS-mCherry
S4d	Rab5-GFP, hTau, mCherry	dHB9-Gal4, hTau, UAS-Rab5-GFP, UAS-mCherry
S5a		GMR-Gal4, UAS-Rab7^{WT}-GFP, UAS-VPS35-HA
S5a		GMR-Gal4, UAS-Rab7^{T22N}-GFP, UAS-VPS35-HA
S5b	Control	GMR-Gal4, UAS-mCD8GFP
S5b	Rab7-YFP	GMR-Gal4, Rab7-YFP/+
S5b	Rab7-YFP, DeGradFP	GMR-Gal4, Rab7-YFP/+, UAS-DeGradFP
S5d	Control	GMR-Gal4, UAS-mCD8GFP
S5d	hTau, GFP	GMR-Gal4, UAS-hTau, UAS-mCD8GFP
S5d	hTau, Rab7^{WT}	GMR-Gal4, UAS-hTau, UAS-Rab7^{WT}
S5d	hTau, Rab7^{Q67L}	GMR-Gal4, UAS-hTau, UAS-Rab7^{Q67L}
S5d	hTau, Rab7^{T22N}	GMR-Gal4, UAS-hTau, UAS-Rab7^{T22N}
S5f	Control	GMR-Gal4, UAS-mCD8GFP

Supplementary Table 1
Expanded figure genotypes

S5f	hTau,Control^{Ri}	GMR-Gal4, UAS-hTau, UAS-mCD8GFP, UAS-Control^{Ri}
S5f	hTau, Rab7^{T22N}	GMR-Gal4, UAS-hTau, UAS-Rab7^{T22N}-GFP, UAS-Control^{Ri}
S5f	hTau, Rab7^{T22N}, Dronc^{Ri}	GMR-Gal4, UAS-hTau, UAS-Rab7^{T22N}, UAS-Dronc^{Ri}
S5h	Control	GMR-Gal4, UAS-mCD8GFP
S5h	hTau^{421X}, GFP	GMR-Gal4, UAS-hTau^{421X}, UAS-mCD8GFP
S5h	Tau^{421X}, Rab7^{T22N}	GMR-Gal4, UAS-Tau^{421X}, UAS-Rab7^{T22N}
S6a	hTau^{D421E}, GFP	GMR-Gal4, UAS-hTau^{D421E}, mCD8GFP
S6a	Tau^{D421E}, Rab7^{T22N}	GMR-Gal4, UAS-hTau^{D421E}, UAS-Rab7^{T22N}
S6b	hTau^{421X}, GFP	GMR-Gal4, UAS-hTau^{421X}, UAS-mCD8GFP
S6b	Tau^{421X}, Rab7^{T22N}	GMR-Gal4, UAS-hTau^{421X}, UAS-Rab7^{T22N}
S6d	hTau, GFP	GMR-Gal4, UAS-hTau, UAS-mCD8GFP
S6d	hTau, Rab7^{WT}	GMR-Gal4, UAS-hTau, UAS-Rab7^{WT}
S6d	hTau, Rab7^{Q67L}	GMR-Gal4, UAS-hTau, UAS-Rab7^{Q67L}
S6f	Control	dTau-Gal4, UAS-mCD8GFP, tubulin-Gal80^{ts}
S6f	Rab7^{T22N}	dTau-Gal4, UAS-mCD8GFP, Rab7^{T22N}, tubulin-Gal80^{ts}
S6f	hTau	dTau-Gal4, UAS-hTau, UAS-mCD8GFP, tubulin-Gal80^{ts}
S6f	hTau, Rab7^{T22N}	dTau-Gal4, UAS-hTau, UAS-Rab7^{T22N}, tubulin-Gal80^{ts}
